# Analyses of internal structures and defects in materials using physics-informed neural networks

**DOI:** 10.1126/sciadv.abk0644

**Published:** 2022-02-16

**Authors:** Enrui Zhang, Ming Dao, George Em Karniadakis, Subra Suresh

**Affiliations:** 1 Division of Applied Mathematics, Brown University, Providence, RI 02912, USA.; 2 Department of Materials Science and Engineering, Massachusetts Institute of Technology, Cambridge, MA 02139, USA.; 3 School of Engineering, Brown University, Providence, RI 02912, USA.; 4 Nanyang Technological University, 639798 Singapore, Singapore.

## Abstract

Characterizing internal structures and defects in materials is a challenging task, often requiring solutions to inverse problems with unknown topology, geometry, material properties, and nonlinear deformation. Here, we present a general framework based on physics-informed neural networks for identifying unknown geometric and material parameters. By using a mesh-free method, we parameterize the geometry of the material using a differentiable and trainable method that can identify multiple structural features. We validate this approach for materials with internal voids/inclusions using constitutive models that encompass the spectrum of linear elasticity, hyperelasticity, and plasticity. We predict the size, shape, and location of the internal void/inclusion as well as the elastic modulus of the inclusion. Our general framework can be applied to other inverse problems in different applications that involve unknown material properties and highly deformable geometries, targeting material characterization, quality assurance, and structural design.

## Teaser

Physics-informed deep learning helps detect unknown internal structures and defects with limited nondestructive measurements.

## INTRODUCTION

Deep learning (*1*) approaches play an increasingly substantial role in a wide range of technologies that benefit computer vision (*2*), natural language processing (*3*), and other data-rich areas of societal interest. Despite the evolving sophistication of data analytics and neural networks (NNs), much of this work to date has not been predicated on a large volume of scientific data, through which predictive models can be constructed using experimentally validated mechanistic inferences and laws of physics. In most scientific applications, by contrast, physical conservation laws (such as those for momentum and energy) are framed by highly general, mathematical formulations [e.g., those invoking partial differential equations (PDEs) in areas such as solid mechanics, fluid mechanics, and material diffusion], along with experimental authentication by recourse to laboratory tests.

Emerging research reveals the profound untapped potential of physics-based, multidisciplinary, deep learning approaches with unprecedented opportunities for scientific and engineering advances in molecular analysis (*4*), design of materials with improved properties and performance (*5*, *6*) in structural and functional applications, and unique pathways for the characterization of properties of materials (*7*–*11*). To further realize this potential, broadly applicable methodologies in the area of NNs are needed to address a variety of issues that underpin deep learning analyses, governed by physical laws and guided by mathematical formulations. To this end, a physics-informed deep learning approach has recently been proposed (*12*) for the simulation of systems governed by physical laws that are represented by PDEs. While traditional methods based on deep learning implicitly encode these formulations by feeding training data governed by equations, this approach explicitly encodes known physical or scaling laws in the form of mathematical equations into the standard structure of NNs, formulating the so-called physics-informed NNs (PINNs) (*12*). Such an approach integrates any existing knowledge expressible in terms of PDEs during the learning process, thereby markedly improving predictability while reducing the amount of data required to achieve a desired level of accuracy. Studies have shown the applicability of PINNs in addressing a wide spectrum of forward and inverse problems spanning disciplines such as fluid mechanics (*13*–*15*), quantum mechanics (*12*), and solid mechanics (*16*–*22*). These applications have shown promise for enhancing predictability when the amount of data is limited or when the problem is ill posed, situations in which existing methods are not likely to yield accurate and reliable results. This approach has been further extended to offer unique pathways to address relevant mathematical formulations, such as stochastic PDEs (*23*) and fractional PDEs (*24*).

Here, we address geometry identification problems in the field of continuum solid mechanics. Geometry identification problems are a class of inverse problems of scientific, technological, and societal interest in fields as diverse as the following (*25*–*27*): safety and failure analysis of civil, mechanical, nuclear, and aeronautical structures; land, sea, and air transportations; reliability analysis in microelectronic devices; nondestructive testing of materials; and processing of engineered materials. In a geometry identification problem, the unknown geometric features and parameters are determined in a solid material/structure given measured material response under static or dynamic loading, thereby characterizing unknown structures including internal defects or boundaries such as voids, vacancies or holes (*28*–*32*), inclusions and reinforcements (*31*, *33*–*36*), and/or cracks (*30*–*32*, *37*). Traditionally, computational algorithms for geometry identification are established on the basis of the finite element method (FEM) (*38*) as the forward solver. Beyond the forward solver, considerable effort is required for the design and implementation of iterative algorithms for updating the estimated values of geometric parameters (*39*) (see section S1 for a brief review of the algorithms), through which the discrepancy (loss) between the observed data and the results of the forward solver is minimized. However, the embedded forward FEM solver as a mesh-based method inherently brings about complications in these algorithms. The estimated geometry is updated by repeatedly remeshing the domain through iterations (*33*). Alternatively, the unknown domain is embedded in a larger fixed domain while introducing an auxiliary field to track the presence of material (*28*, *36*, *40*). The problem becomes even more challenging when large deformations (i.e., geometric nonlinearity) and nonlinear mechanical properties (i.e., highly nonlinear constitutive behavior of the solid material) are involved. These issues are still not well resolved, and available methods are cumbersome and resource intensive for deriving automated solutions to such inverse problems involving unknown geometry.

**Fig. 1.**
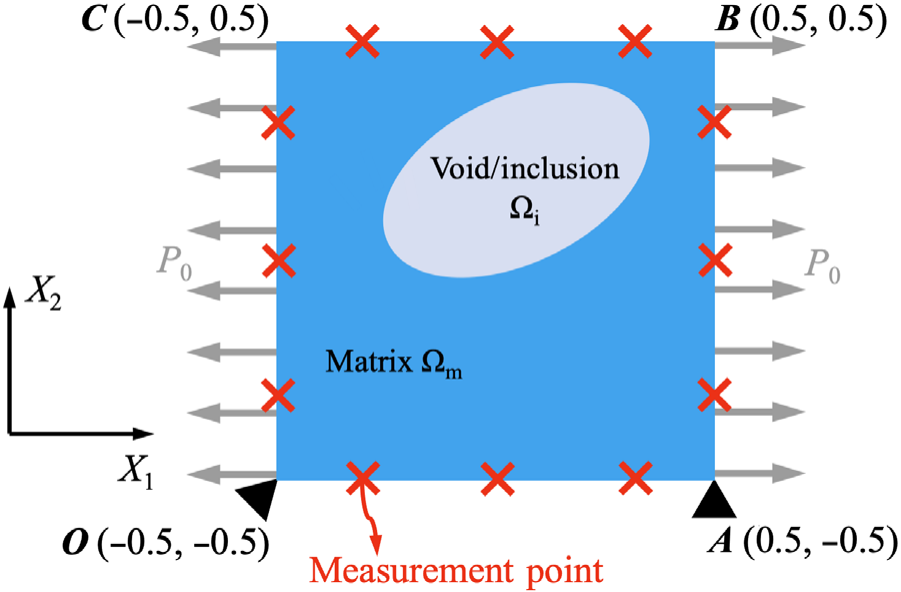
General setup of the prototypical problem on geometry and material identification in this study. We consider a plane-strain problem in the *X*_1_-*X*_2_ plane about a square-shaped matrix specimen Ω_m_ with a void/inclusion Ω_i_. Displacements are measured on the outer boundary of the matrix when loading *P*_0_ is applied. The goal is to characterize the unknown geometry of the internal void/inclusion according to the measurement data. For the case of inclusion, material properties of the inclusion are also characterized.

Here, we present a unique, systematic approach based on PINNs for solving geometry identification problems in continuum solid mechanics. This method integrates known PDEs of importance in solid mechanics with NNs, composing a unified computational framework involving both the forward solver and the inverse algorithm. Notably, we propose a method for directly parameterizing the geometry of the solid in a differentiable and trainable manner. By using the workflow of NNs, our method can automatically update the geometry estimation through the deep learning process. To demonstrate the efficacy of our method, we study a two-dimensional prototypical problem on a matrix-void/inclusion system as a proof of concept (see Fig. 1). A square-shaped matrix material contains a void/inclusion with unknown geometry. To characterize the location, size, and shape of the void/inclusion, we apply loading *P*_0_ on the matrix boundary and monitor the displacement response on the measurement points at the matrix boundary under such loading. We expect the PINN to inversely characterize the geometry of the void/inclusion according to the displacement data. To test the performance of our method with various parametric assessments, we build a set of detailed cases for this problem, including different shapes and topologies of the void and different constitutive models for describing the mechanical properties of the material. For the particular case of inclusion, the PINN is also required to estimate the unknown material parameter of the inclusion, through which we demonstrate the capability of our model in solving combined material and geometry identification problems. In addition to the major results shown in the main text, we report in the Supplementary Materials more systematic studies of additional cases and parametric analyses, highlighting the advantages and limitations of the method.

## RESULTS

### Setup of the prototypical inverse problem

**Fig. 2.**
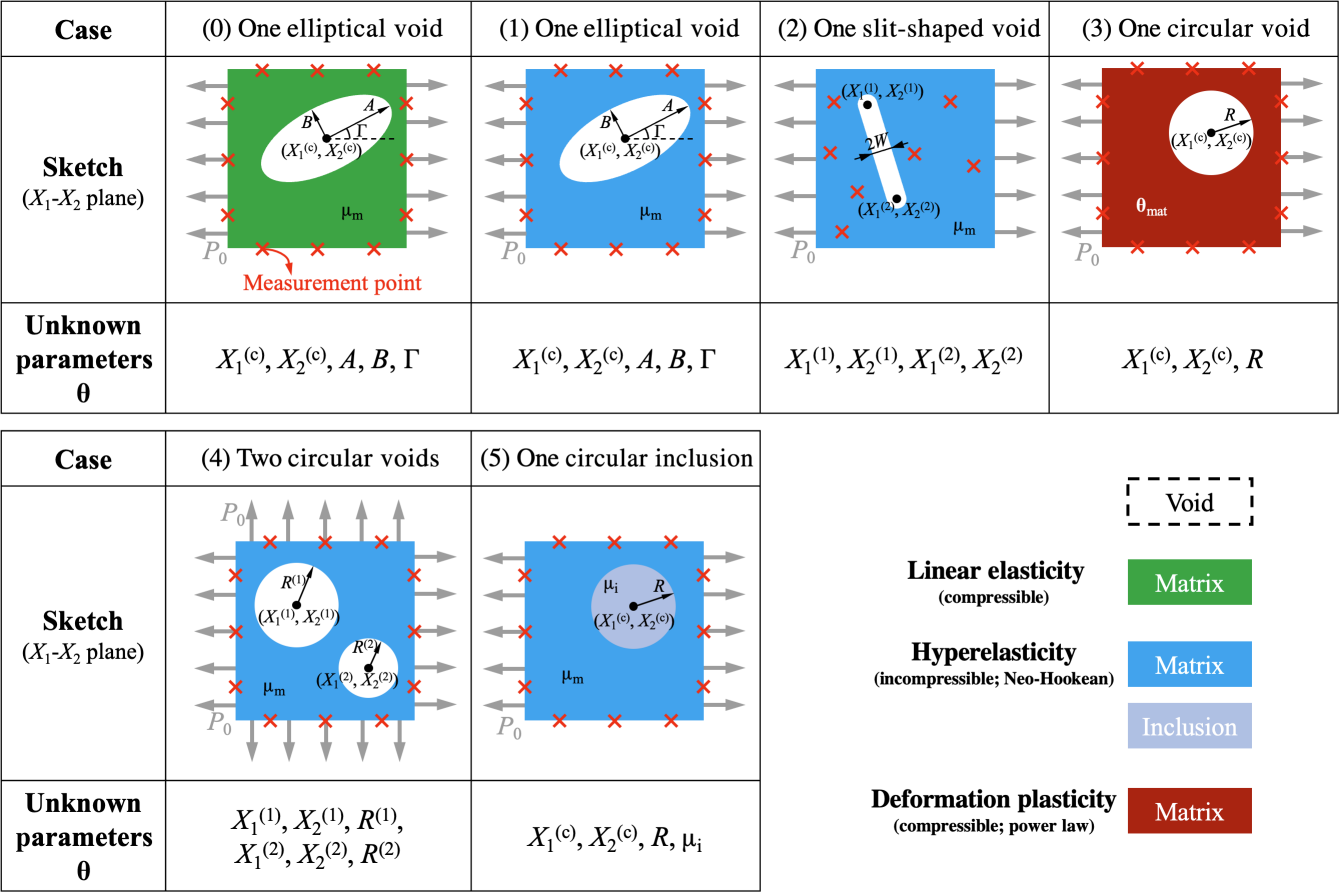
Setup of cases 0 to 5 of the prototypical problem. All the illustrations and geometric parameters are given in the reference (undeformed) configuration. The target of each case is to estimate the unknown parameters **θ** given the displacement data on the measurement points. All unknown parameters describe the geometry of the void/inclusion except μ_i_ in case 5, which is the shear modulus of the inclusion. For each case, we specify the type of the inhomogeneity (void/inclusion), unknown parameters, the material model (linear elasticity/hyperelasticity/deformation plasticity), type of the loading (uniaxial/biaxial), and the location of displacement measurements (uniformly on the outer boundary/inside the solid). Additional cases are summarized in the main text and presented in detail in the Supplementary Materials.

The general setup of the prototypical inverse problem has been presented in the introduction and in Fig. 1. We consider a plane-strain problem in the *X*_1_ − *X*_2_ plane about a square-shaped matrix specimen with a void/inclusion. The goal of the inverse problem is to estimate the geometric parameters **θ**_geo_ (and material parameters **θ**_mat_ in the constitutive model) of the void (inclusion) Ω_i_ inside the matrix Ω_m_, by applying uniaxial/biaxial loading *P*_0_ and collecting displacement data on the matrix boundary. We designed six specific plane-strain problems as shown in Fig. 2. For each case, we specify the type of the inhomogeneity (void/inclusion), the unknown parameters [**θ**_geo_ for void, or **θ**_geo_ and **θ**_mat_ for inclusion; denoted together as **θ** = (**θ**_mat_, **θ**_geo_)], the material model (compressible linear elasticity, incompressible Neo-Hookean hyperelasticity, or compressible deformation plasticity), type of the loading (uniaxial/biaxial), and the location of displacement measurements (uniformly on the outer boundary/inside the solid). All unknown parameters describe the geometry of the void/inclusion except μ_i_ in case 5, which is a material parameter representing the shear modulus of the inclusion. The sketch and all the geometric parameters are shown in the reference (undeformed) configuration. The material properties of the matrix are known for all the cases.

The solution of the six cases will provide a proof of concept for our method under different practical scenarios, demonstrating the wide applicability of the method. The three material models (cases 0, 1, and 3 as the baseline cases) cover a wide range of mechanical behavior patterns of natural and engineered materials in a vast array of practical applications. We place the displacement measurement points only on the outer boundary of the matrix, to mimic the real-world situation where the internal details are not available. Case 2 explores the scenario of engineering application where the void has a large aspect ratio (such as a crack), which we approximate by a slender slit. For this case only, we allow the displacement measurements to be inside the solid because of the relative insensitivity of the boundary displacement with respect to the slit geometry. Case 4 demonstrates the applicability of the method for materials with multiple voids (such as porous materials or those with multiple cracks/slits). Last, for case 5, we estimate the material and geometric parameters for a soft circular inclusion, to show that our method can handle combined material and geometry identification problems.

### Summary of PINN architecture for continuum solid mechanics

**Fig. 3.**
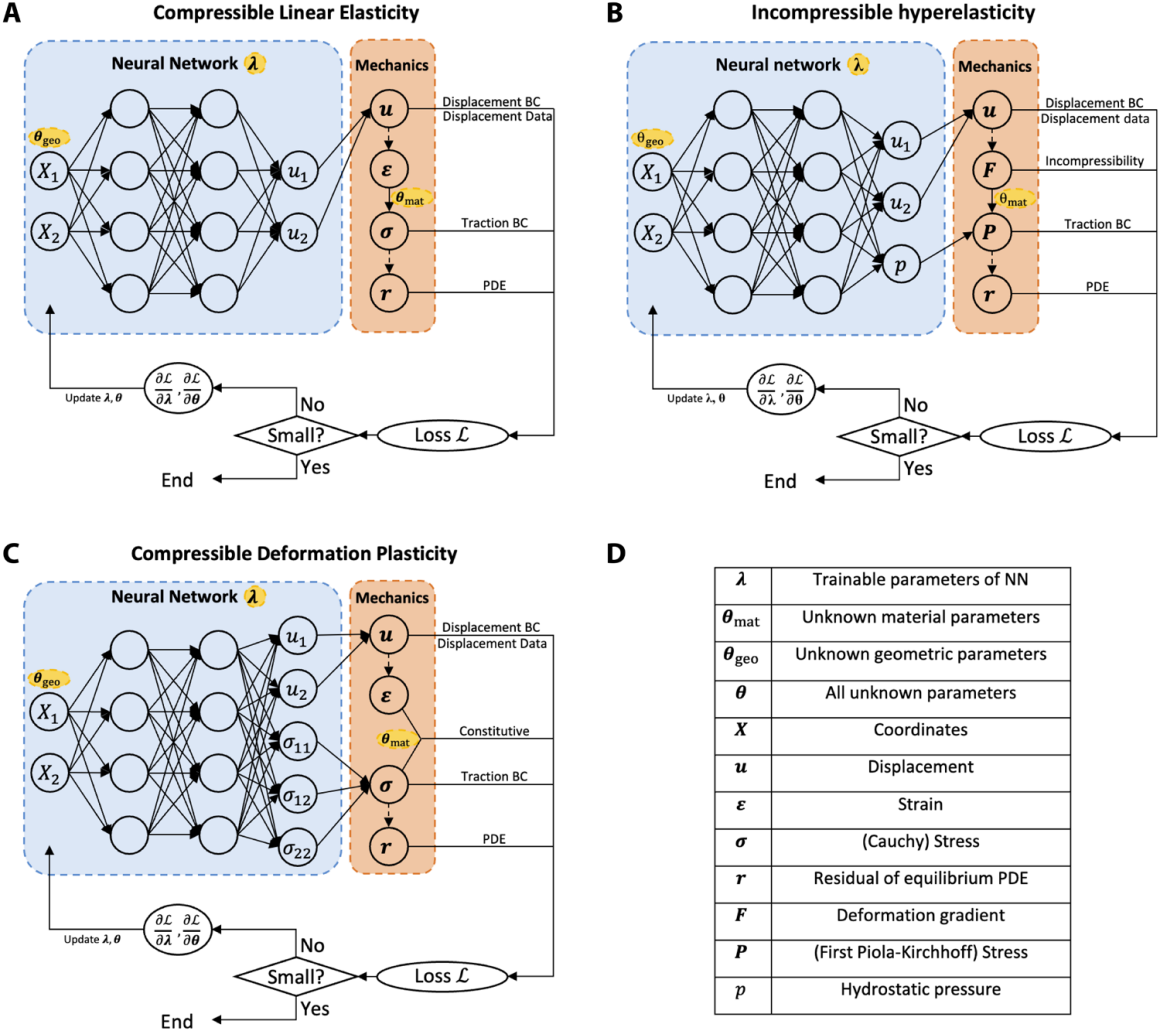
Architectures of PINNs for continuum solid mechanics. We established the PINNs for plane-strain problems involving geometry and material identification. Three material models are considered, including (**A**) compressible linear elasticity, (**B**) incompressible hyperelasticity, and (**C**) deformation plasticity. (A to C) We apply NNs with trainable parameters λ to approximate primary solution fields with respect to the in-plane coordinates (*X*_1_, *X*_2_). Mechanical laws are integrated to derive relevant mechanical quantities of interest from the NN outputs, such as strain, stress, and the residual of equilibrium PDEs, during which unknown material parameters **θ**_mat_ are involved. The loss function ℒ is formulated to represent the prediction error of each condition in the problem, such as PDEs, BCs, and data in (A), during which unknown geometric parameters **θ**_geo_ are involved because of the variable computational domain. Last, parameter estimation is conducted through the minimization of loss function. In this process, λ and **θ** = (**θ**_mat,_ **θ**_geo_) are iteratively updated. The final solution of the identification problem is the updated value of **θ** after iterations. (**D**) Definitions of the notations in (A) to (C).

We set up the general formulation of PINNs in continuum solid mechanics involving both material and geometry identification. Corresponding to our computational examples, we design the architectures of the PINNs for plane-strain problems for the three material models, as shown in (i) Fig. 3A for (compressible) linear elasticity, (ii) Fig. 3B for (incompressible) hyperelasticity, and (iii) Fig. 3C for (compressible) deformation plasticity. The architectures of the PINNs are slightly different for different material models because of the characteristics of their mathematical expressions. Figure 3D includes the definitions of the mechanical quantities of interest in the architectures.

**Fig. 4.**
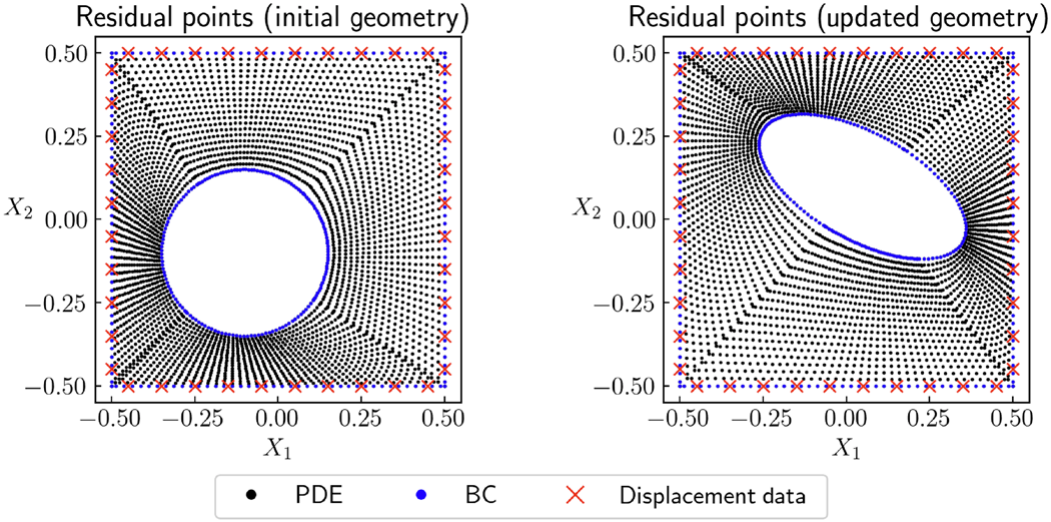
Residual points for the initial geometry and updated geometry in case 0. Different residual terms (PDEs, BCs, and data) require different residual points. We propose the geometry-parameterized residual points, so that the locations of the residual points automatically change as the geometric parameters **θ**_geo_ are updated.

The detailed formulations and relevant governing equations of PINNs are explained in Materials and Methods and in section S2. Here, we summarize the basic workflow of PINNs as follows. First, we apply an NN (with trainable parameters **λ**) to approximate the primary solution fields (top left panels in Fig. 2, A to C) with respect to the in-plane coordinates **X** = (*X*_1_, *X*_2_). Second, we integrate the mechanical laws into the PINN architecture (top right panels in Fig. 2, A to C) by deriving relevant mechanical quantities of interest from the NN outputs, such as strain, stress, and the residual of equilibrium PDEs. In this process, unknown material parameters **θ**_mat_ are involved. Third, we formulate the loss function ℒ(**λ**, **θ**), which measures the discrepancy between the predicted mechanical quantities of interest and their respective true values provided by mechanical laws and measured data (bottom right panels in Fig. 2, A to C). For example, for linear elasticity in Fig. 2A, the loss function is expressed as

$$L(\lambda ,\theta )=\alpha_{\text{PDE}}L_{\text{PDE}}(\lambda ,\theta )+\alpha_{\text{BC}}L_{\text{BC}}(\lambda ,\theta )+\alpha_{\text{data}}L_{\text{data}}(\lambda ,\theta )$$ (1)

where the three loss terms ℒ*_j_*(**λ**, **θ**) (*j* = PDE, BC, and data) weighted by α*_j_* correspond to PDEs, boundary conditions (BCs), and data, respectively. Each loss term is the mean squared error evaluated on *N_j_* residual points

$$L_{j}(\lambda ,\theta )=\frac{1}{N_{j}}\sum_{i=1}^{N_{j}}{|r_{j}(X_{j}^{\left(i\right)}(\theta_{\text{geo}});\lambda ,\theta_{\text{mat}})|}^{2}$$ (2)

where **r***_j_* is the residual of the condition *j* at the *i*th residual point $X_{j}^{\left(i\right)}(\theta_{\text{geo}})$. The *N_j_* residual points are distributed in the domain of condition *j* to correctly evaluate ℒ*_j_*(**λ**, **θ**). As an example, we show the residual points for each condition in case 0 before and during the simulation in Fig. 4. The coordinates of the residual points $X_{j}^{\left(i\right)}$ depend on geometric parameters **θ**_geo_ because of the variable computational domain, which will be explained in detail in the next section. Last, we conduct parameter estimation through the training of the PINN (bottom left panels in Fig. 2, A to C), during which the estimated unknown parameters **θ** = (**θ**_mat_, **θ**_geo_) and NN parameters **λ** are updated/trained to minimize the loss function. This process can be expressed as

$$\hat{\lambda },\hat{\theta }=\underset{\lambda ,\theta }{\text{argmin}}L(\lambda ,\theta )$$ (3)

where the hat symbol refers to the value of these trainable parameters after the training process is completed. As the solution to the inverse problem, the estimation of the unknown parameters is $\hat{\theta }$.

### Formulation for geometry identification

Geometric parameters **θ**_geo_ play an essentially different role in the inverse problem compared to material parameters **θ**_mat_. Material parameters parameterize the governing PDEs of mechanics, which are naturally endowed with trainability through automatic differentiation of (physics-informed) NNs. As a result, material parameters can be directly estimated using the standard formulation of PINNs for inverse problems (*12*, *18*, *41*, *42*). Geometric parameters **θ**_geo_, on the other hand, parameterize the computational domains of the PDEs and BCs, which do not naturally serve as trainable parameters in the framework of PINNs. To make the geometric parameters **θ**_geo_ differentiable and hence trainable in a similar way to material parameters **θ**_mat_, we propose to parameterize the coordinates of residual points by geometric parameters **θ**_geo_. Technically, such parameterization can be implemented by using the definition of trainable variables in deep learning libraries such as TensorFlow (*43*): We first define the geometric parameters **θ**_geo_ as trainable variables; then, we express the locations of residual points as functions of these trainable variables. As a result, the coordinates of residual points are automatically updated as the estimation of **θ**_geo_ is updated throughout the iterative training process (see Fig. 4). In this way, we ensure that the residual points for different conditions are always located in their correct domains. Furthermore, this implementation allows us to capture the gradient of the loss function ℒ with respect to the geometric parameters **θ**_geo_, which otherwise could not be realized using the standard formulations of PINNs (*44*, *45*). With the geometry-parameterized residual points, the PINN can correctly update the geometric parameters **θ**_geo_ throughout the training process, thereby characterizing the unknown geometry. To the best of our knowledge, such form of parameterization invoking PINNs to solve geometry identification problems has hitherto not been addressed.

### Procedure of simulation

We adopted Abaqus (*46*) as the finite element solver to generate the computational examples. Specifically, we preset reference values of unknown parameters to be θ* and conducted forward simulations, which generated the displacement data provided to the PINN and ground-truth full-field solution for assessing the performance of the PINN. The PINN initialized the estimation of unknown parameters to be **θ**^0^. The PINN first went through a pretraining procedure for stabilizing the forward prediction, where the estimated parameters were fixed to be **θ**^0^ (see Materials and Methods for details). As the PINN initiated parameter estimation through the iterative training process, we expected that the estimated parameters **θ** would migrate toward the correct value θ*. The training process terminated after the loss function and the estimated parameters reached a relative plateau, yielding the parameter estimation results $\hat{\theta }$. The detailed setups of the prototypical problem, the finite element solver, and the hyperparameters of the PINN are included in section S3.

We present the major results for cases 0 to 5 in the main text. Further results for cases 0 to 5 are included in section S4 (figs. S1 and S2). To justify our choice of hyperparameters of the PINN (width and depth of the NN, weights of loss components, and number of residual points), we show the results of a parametric study for a forward problem in sections S5 and S6 (figs. S3 to S5). In addition, we consider other modified setups of our inverse problem in sections S7 to S11 (figs. S6 to S9 and table S1) for illustrating the applicability, characteristics, and limitations of our method for the prototypical problems. For these additional cases, we summarize the objectives and major findings in the following sections and present the detailed results in the Supplementary Materials.

### Parameter estimation results

The results of parameter estimation for cases 0 to 5 are shown in Table 1. For each case, we compare the estimated and reference values of the unknown parameters by presenting absolute errors and relative errors. To calculate the relative error, we normalize the coordinates, the lengths and the modulus, and the tilting angle by the domain size (side length of the matrix), respective reference values, and 180°, respectively. Table 1 indicates that the PINN estimates unknown parameters with high accuracy, with relative error O(10^−2^) on most parameters and as small as O(10^−4^) for some parameters.

**Table 1.** Parameter estimation for all cases shown in Fig. 2. We compare the estimated values ($\hat{\theta }$) and reference values (θ*) of unknown parameters. To calculate the relative error, we normalize the coordinates, the lengths and the modulus, and the tilting angle by the domain size (side length of the matrix), their respective reference values, and 180°, respectively. To improve the accuracy of case 5, we provide the PINN with additional displacement measurement points inside the solid and then retrain the PINN, which is shown in the table as “Case 5 (with internal data)”.

Case 0	$X_{1}^{\left(c\right)}$	$X_{2}^{\left(c\right)}$	*A*	*B*	Γ	
Estimated value	0.0488	0.0987	0.3475	0.1582	−29.42°		
Reference value	0.05	0.10	0.35	0.15	−30°		
Absolute error (× 10^−2^, except Γ)	0.12	0.13	0.25	0.82	0.58°		
Relative error (%)	0.12	0.13	0.71	5.47	0.32		
**Case 1**	$X_{1}^{\left(c\right)}$	$X_{2}^{\left(c\right)}$	*A*	*B*	Γ		
Estimated value	0.0479	0.0991	0.3440	0.1602	−29.02°		
Reference value	0.05	0.10	0.35	0.15	−30°		
Absolute error (× 10^−2^, except Γ)	0.21	0.09	0.60	1.02	0.98°		
Relative error (%)	0.21	0.09	1.7	6.8	0.54		
**Case 2**	$X_{1}^{\left(1\right)}$	$X_{2}^{\left(1\right)}$	$X_{1}^{\left(2\right)}$	$X_{2}^{\left(2\right)}$			
Estimated value	−0.0399	0.3273	0.0396	−0.2315				
Reference value	−0.0392	0.3474	0.0392	−0.2474				
Absolute error (× 10^−2^)	0.07	2.01	0.04	1.59				
Relative error (%)	0.07	2.01	0.04	1.59				
**Case 3**	$X_{1}^{\left(c\right)}$	$X_{2}^{\left(c\right)}$	*R*					
Estimated value	0.0506	0.0999	0.2525						
Reference value	0.05	0.10	0.25						
Absolute error (× 10^−2^)	0.06	0.01	0.25						
Relative error (%)	0.06	0.01	1.00						
**Case 4**	$X_{1}^{\left(1\right)}$	$X_{2}^{\left(1\right)}$	*R* ^(1)^	$X_{1}^{\left(2\right)}$	$X_{2}^{\left(2\right)}$	*R* ^(2)^
Estimated value	−0.15089	0.10018	0.20007	0.25045	−0.05008	0.15019
Reference value	−0.15	0.10	0.20	0.25	−0.05	0.15
Absolute error (× 10^−2^)	0.089	0.018	0.007	0.045	0.008	0.019
Relative error (%)	0.089	0.018	0.04	0.045	0.008	0.13
**Case 5**	$X_{1}^{\left(c\right)}$	$X_{2}^{\left(c\right)}$	*R*	μ_i_			
Estimated value	0.0496	0.0991	0.2583	0.0760				
Reference value	0.05	0.10	0.25	0.0667				
Absolute error (× 10^−2^)	0.04	0.09	0.83	0.93				
Relative error (%)	0.04	0.09	3.3	13.9				
**Case 5 (with internal data)**	$X_{1}^{\left(c\right)}$	$X_{2}^{\left(c\right)}$	*R*	μ_i_				
Estimated value	0.0495	0.0998	0.2524	0.0687				
Reference value	0.05	0.10	0.25	0.0667				
Absolute error (× 10^−2^)	0.05	0.02	0.24	0.20				
Relative error (%)	0.05	0.02	0.96	3.0				

It is worth noting that the estimated shear modulus of the inclusion μ_i_ in case 5 has an error slightly more than 10%. We provide a discussion on this issue in a following section (“Interpreting the convergence histories”). To improve the accuracy of case 5, we suppose that five additional data points inside the solid are available as in case 2. We retrain the PINN with the expanded measurement data and append the results in Table 1 as the modified case 5. With the additional data, the relative error of estimated parameters decreases to O(10^−2^), similar to other cases. In summary, given scattered displacement measurements, the PINN can accurately characterize the geometry (and material properties) of the internal void(s)/inclusion for various problem setups, including different constitutive relations, shapes of voids, and numbers of voids. The result indicates the generality of our method for solving a broad spectrum of inverse problems in mechanics of materials.

In sections S7 to S10, we provide additional parametric studies based on simplified cases 1 and 5 for demonstrating the influence of various factors on the estimation accuracy of unknown parameters, including the locations of measurement points (S7), the size of the void (S8), the location of the void (S9), and the moduli ratio of matrix and inclusion (S10). These studies show that our method is robust against various choices of true values θ*, including different locations and different sizes [no smaller than O(10^−1^) of the matrix geometry] of the void, and moduli ratio of matrix and inclusion spanning within roughly O(10^1^). In addition, without prior knowledge on the location of the void, the measurement points should be uniformly placed on the matrix boundary, to make sure that the displacement data effectively capture the key information related to the void/inclusion.

### Inference of deformed patterns

**Fig. 5.**
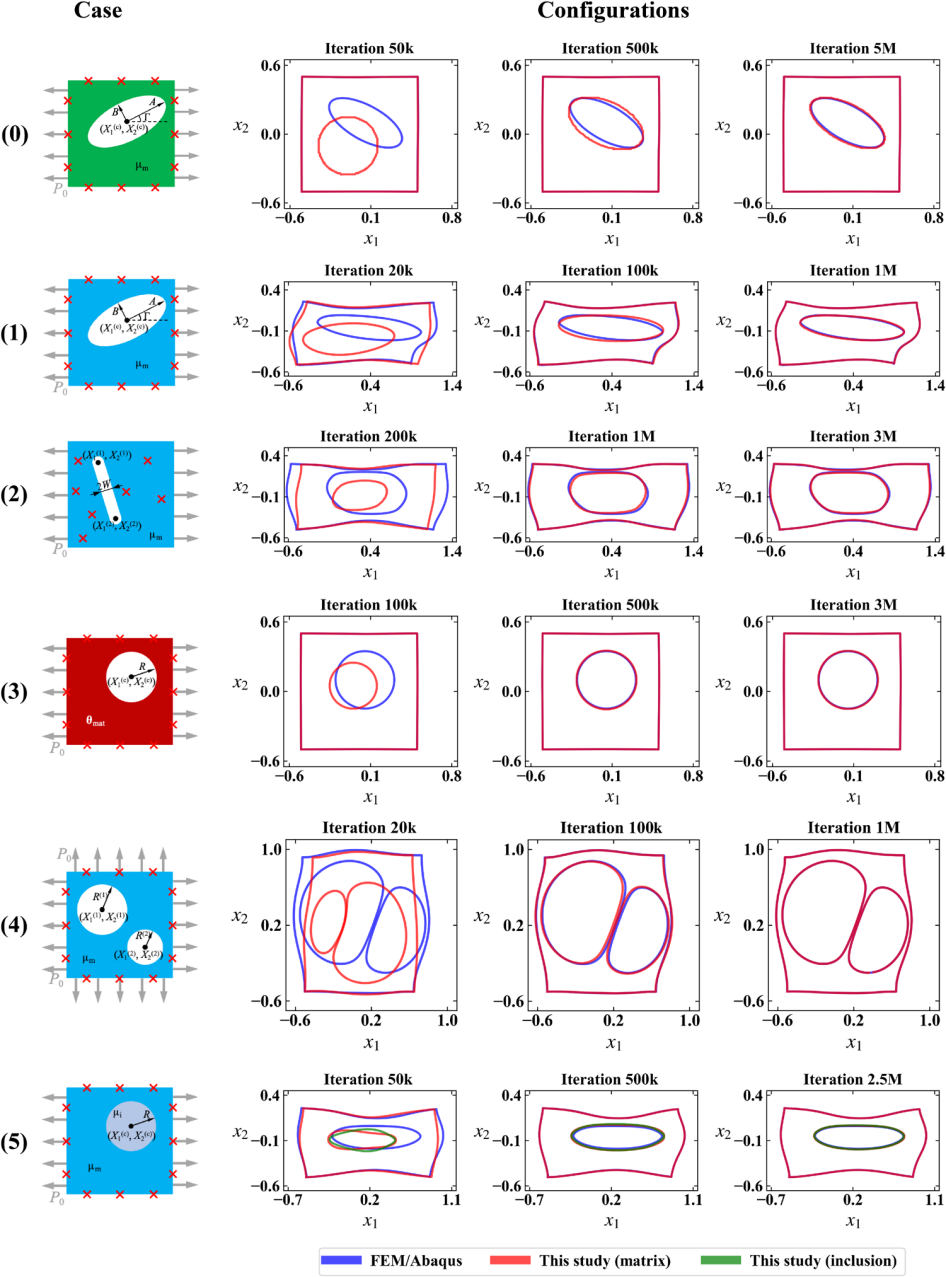
Inference of the deformed patterns compared with FEM ground truth for all cases shown in Fig. 2. We displayed the visual outlines of deformed configurations of FEM/Abaqus (blue) and PINN results (red for matrix; green for inclusion in case 5). Three snapshots are shown for each case after different numbers of training iterations, which (from left to right) correspond to the completion of pretraining (beginning of parameter estimation), amid the training, and the completion of training, respectively (k = 10^3^ and M = 10^6^).

Our method not only is capable of estimating unknown parameters but also provides quantitative measures of the deformed patterns of the solid. Specifically, we apply the estimation results $\hat{\lambda }$ and ${\hat{\theta }}_{\text{geo}}$ (see Eq. 3) to the NN part of the PINN (top left panels in Figs. 2, A to C) to infer the deformed configuration, where ${\hat{\theta }}_{\text{geo}}$ determines the reference/undeformed configuration and $\hat{\lambda }$ determines the mapping from the reference/undeformed configuration to the deformed configuration. In Fig. 5, we display the comparison of the deformed configurations between the FEM ground truth (blue) and the PINN inference results (red for matrix; green for inclusion in case 5) for the six cases. Three snapshots are shown for each case after different numbers of training iterations (*k* = 10^3^ and *M* = 10^6^), which from the left to the right correspond to the completion of pretraining (beginning of parameter estimation), amid the training, and the completion of training, respectively. For clarity of presentation, this figure shows the outer and inner boundaries of the specimen visualized from the FEM and PINN analyses. In the snapshots in the second column, the two outlines match each other to a high extent. The remaining minor discrepancy gradually diminishes through the remaining iterations. After the training process is completed, the deformed configurations from the PINN are almost identical to those from the FEM ground truth. For case 5, specially, the inner boundary of the matrix (red) and the boundary of the inclusion (green) predicted by the PINN also overlap well with each other, indicating that the continuity of the material surfaces in the matrix-inclusion system is preserved in the inference of the PINN.

**Fig. 6.**
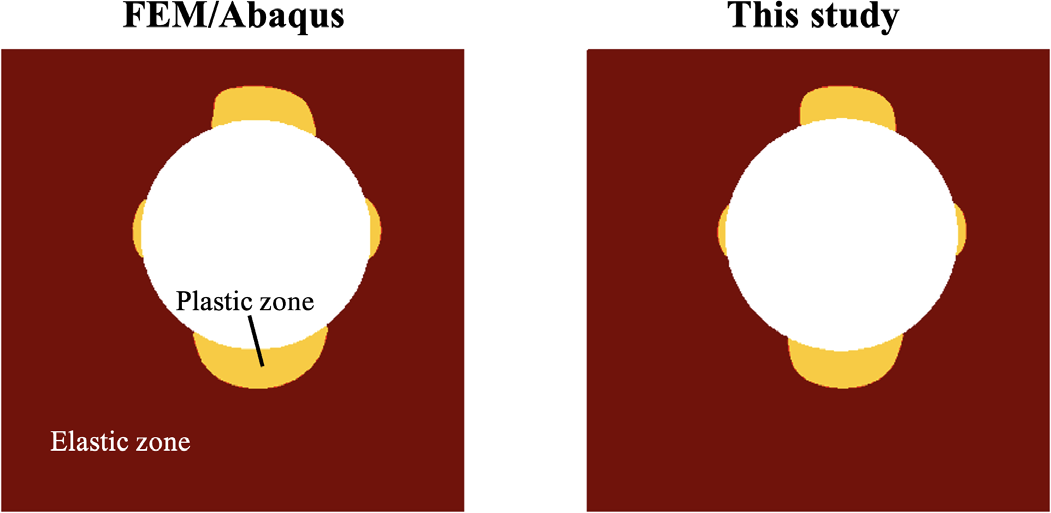
Inference of the plastic zone compared with FEM ground truth in case 3 shown in Fig. 2. We mark the plastic zone by yellow and the void by white.

For case 3 where plasticity is involved, we also examined the inference of the plastic zone. Figure 6 shows the comparison of the plastic zone between the PINN prediction and the FEM ground truth. Not only is the geometry of the void characterized correctly (white region within the matrix) as previously verified in Table 1 and Fig. 5 but also the plastic zone of the loaded matrix is inferred with high accuracy.

### Interpreting the convergence histories

**Fig. 7.**
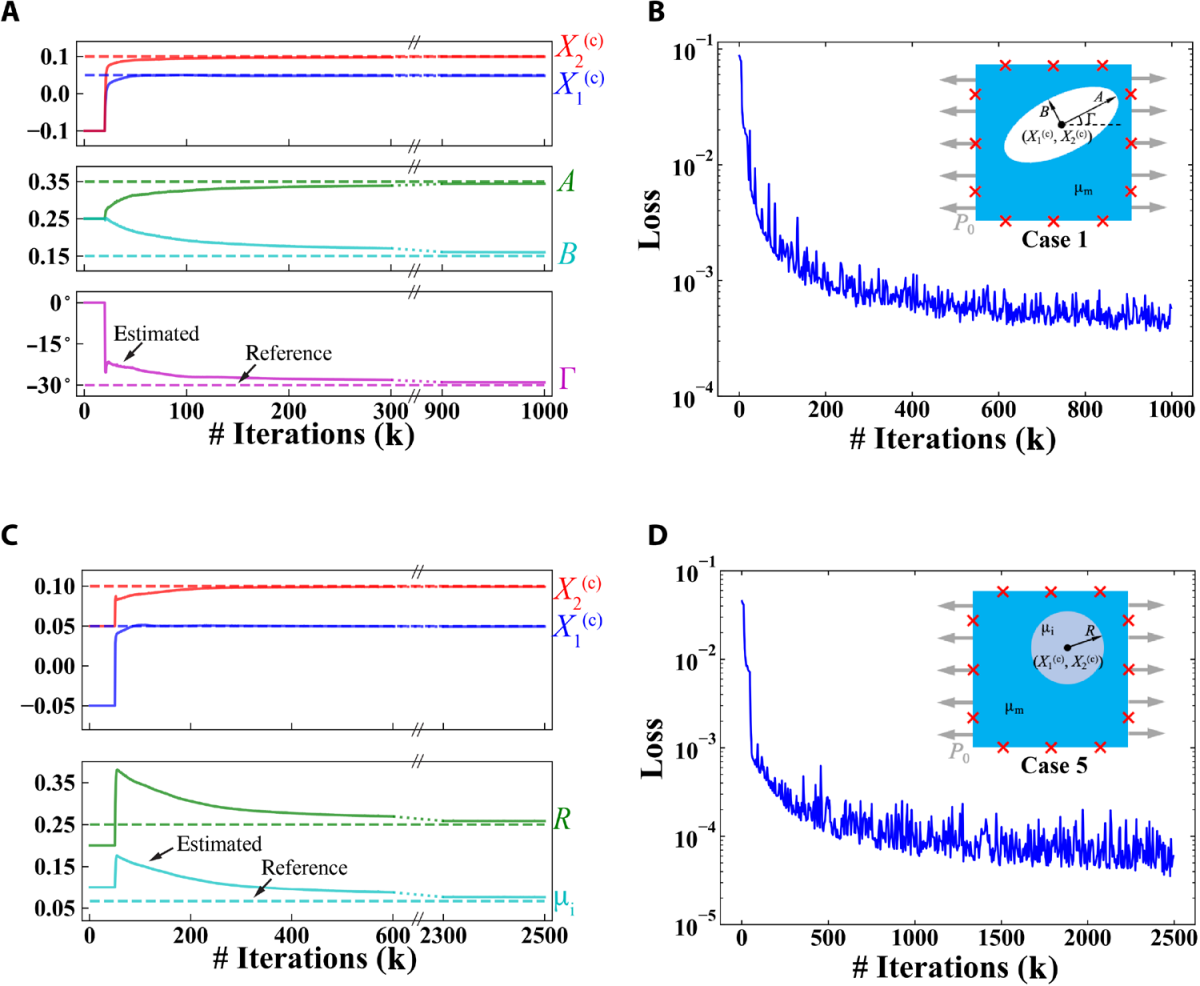
Evolution of estimated unknown parameters and loss function in case 1 and case 5 during the training process. Case 1 (**A** and **B**) involves geometry identification, while case 5 (**C** and **D**) involves both material and geometry identification. See Fig. 2 for the definitions of the cases. (A and C) The dashed lines and solid lines represent the reference value and estimated value of unknown parameters. Unknown parameters are not updated in the pretraining process during the first 20k (for case 1) and 50k (for case 5) iterations, respectively. (B and D) The value of the loss function during the training process. The results for cases 0, 2, 3, and 4 are shown in section S4 (fig. S1).

Besides the final results obtained for parameter estimation and inference of deformation, we also address how the estimated values evolve toward the reference values during the training process. In Fig. 7, we consider case 1 (Fig. 7, A and B) and case 5 (Fig. 7, C and D) as representative examples and show the convergence process for the estimated parameters (Fig. 7, A and C) and loss function (Fig. 7, B and D). The same figures for other cases are included in section S4 (fig. S1). Figure 7A shows the evolution of the estimated values of unknown parameters (solid lines) of case 1 during the training process. As the estimated values become trainable after the initial 20k iterations of pretraining (see Materials and Methods), they rapidly deviate from their respective initial guesses and gradually approach the reference values (dashed lines). After around 300k iterations, the estimations already approach the reference value. Within the remaining 700k iterations, the estimated values further approach the reference values slowly, reaching a high estimation accuracy at the end. Figure 7B shows the evolution of the loss function during the training process. The loss decreases from O(10^−1^) to O(10^−4^). Such a small value of the loss function indicates that all the conditions involved in the loss function are approximately satisfied by the PINN prediction. Similar to the evolution of estimated parameters, the loss decays rapidly at the early stage of training [from O(10^−1^) to O(10^−3^) within 200k iterations]. The rate of decrease turns to be substantially slower during the late stage. We find similar tendency for case 5 in Fig. 7 (C and D) on the evolution of estimated parameters and loss function.

Notably, we find that both the convergence rate and the estimation accuracy differ among the unknown parameters. For cases 1 and 5, the location of the void/inclusion described by $\left(X_{1}^{\left(c\right)},X_{2}^{\left(c\right)}\right)$ almost converges with high accuracy after around 150k/300k iterations, while the remaining unknown parameters exhibit worse behavior in terms of both convergence rate and estimation accuracy, including (*A*, *B*, Γ) in case 1 and (*R*, μ_i_) in case 5. We attribute these two phenomena to the sensitivity of ℒ_data_ with respect to estimated unknown parameters, or the identifiability of unknown parameters. $\left(X_{1}^{\left(c\right)},X_{2}^{\left(c\right)}\right)$ remarkably influence the displacement pattern at the outer boundary. Hence, a small deviation of estimated $\left(X_{1}^{\left(c\right)},X_{2}^{\left(c\right)}\right)$ from their reference values causes a large increase of ℒ_data_. By examining the displacement data, one may even roughly estimate $\left(X_{1}^{\left(c\right)},X_{2}^{\left(c\right)}\right)$ by intuition. On the other hand, a significantly smaller increase of ℒ_data_ is rendered for certain combinations of perturbation on (*A*, *B*, Γ) in case 1 and (*R*, μ_i_) in case 5.

**Fig. 8.**
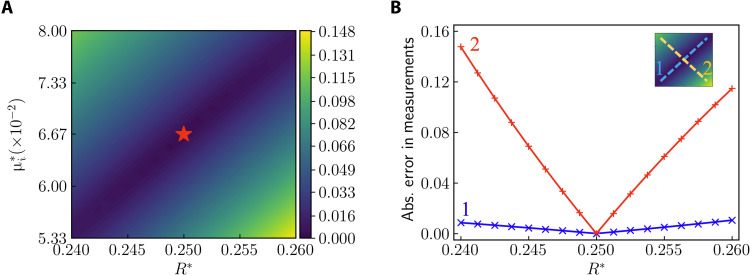
Perturbation analysis of unknown parameters in case 5. Using the finite element solver, we calculate the root mean squared error of the displacement data on the measurement points caused by the perturbation $\left(\Delta R*,\Delta \mu_{i}^{*}\right)$ on the reference values of the shear modulus $\left(\mu_{i}^{*}\right)$ and the radius of the inclusion (R*). (**A**) Absolute error of displacement data for various perturbations on $\left(R*,\mu_{i}^{*}\right)$. The red star at the center corresponds to the unperturbed state $\left(\Delta R*=\Delta \mu_{i}^{*}=0\right)$. (**B**) Absolute error of measurements along the diagonal lines of the $\left(R*,\mu_{i}^{*}\right)$ domain in (A). Perturbation states on line 1 satisfy $\Delta R*\Delta \mu_{i}^{*}>0$. Perturbation states on line 1 satisfy $\Delta R*\Delta \mu_{i}^{*}<0$.

To support our statement on the cause of different convergence rates and estimation accuracies, we use the FEM solver to analyze how a perturbation $\left(\Delta R^{*},\Delta \mu_{i}^{*}\right)$ on the reference values $\left(R^{*},\mu_{i}^{*}\right)$ influences the displacement data collected on the measurement points in case 5. We show the root mean squared error of the displacement data caused by various combinations of $\left(\Delta R^{*},\Delta \mu_{i}^{*}\right)$ in Fig. 8. From Fig. 8A, we observe that the error is significantly smaller for certain combinations of $\Delta R^{*}\Delta \mu_{i}^{*}>0$ than for $\Delta R^{*}\Delta \mu_{i}^{*}<0$. A detailed comparison along the two diagonal lines of the $\left(R^{*},\mu_{i}^{*}\right)$ domain in Fig. 8A is displayed in Fig. 8B. The error along line 1 is roughly O(10^−1^) the error along line 2. Such a phenomenon indicates that ℒ_data_ is insensitive to perturbations satisfying $\Delta R^{*}\Delta \mu_{i}^{*}>0$. As the PINN estimates unknown parameters by minimizing the loss function, there exists intrinsically poor identifiability due to the coexistence of *R* and μ_i_ as unknown parameters and the placement of measurement points on the outer boundary. Such an interaction of *R* and μ_i_ has a twofold effect: First, the accurate estimation of *R* and μ_i_ is postponed to rather late stages of the training process when the total loss has been relatively small; second, the estimation error of *R* and μ_i_ is notably larger than other unknown parameters. Such an analysis matches our observation in Fig. 7C, where many more iterations are needed to estimate *R* and μ_i_. The issue of poor identifiability in case 5 may be mitigated by providing a small number of additional internal data points as shown in Table 1 (see section S4 and fig. S2 for complete results). We conclude that the interplay between the unknown parameters and available data measurements renders relatively poor identifiability for some unknown parameters.

## DISCUSSION

Inverse problems, especially those related to geometry identification, are notoriously difficult to solve for solids with nonlinear constitutive relations. With the hyperelastic solid undergoing severe distortion, it is hard to recover the unknown reference geometry with limited data. By examining the deformed pattern of the void in case 2 (see Fig. 5), it is intuitively not a straightforward task to trace back to the original slit in the reference configuration. In this work, we have demonstrated the capability of PINNs in effectively solving the geometry and material identification problems for engineering solids that incorporate large deformation response and plasticity through our computational examples for the matrix-void/inclusion system. We have shown that the present framework is able to accurately estimate the unknown geometric and material parameters with a relative error O(10^−2^) when proper displacement data are supplied to ensure identifiability.

The approach presented here has some unique characteristics, endowing this method with some distinct advantages. It provides a unified framework for solving forward problems and inverse problems with unknown parameters in PDEs (material identification) and/or domains (geometry identification), by combining the underlying mechanical principles and data into a deep NN. Unlike traditional methods based on FEM, there is neither the need to design problem-specific algorithms to update estimated unknown parameters beyond the forward solver nor the need to repeatedly remesh the computational domain throughout the iterations. In our method, the update of geometry is realized by the automated process built in the deep learning algorithms. In particular, the estimation of geometric parameters is automatically updated as the PINN seeks to minimize the loss function through the iterative training process. With deep learning libraries such as TensorFlow (*43*), the entire length of our PINN code for the current work is merely a few hundred lines. From the perspective of both design and implementation of the algorithm, PINNs reduce the human effort and related costs in setting up algorithms for inverse problems. On the other hand, compared to typical data-driven deep learning approaches, PINNs have the advantage of using well-established mechanics formulations as training guidelines, thereby requiring data only for the current instance of the problem setup and ensuring data efficiency.

Throughout this work, we adopt the Adam optimizer (*47*) as the optimization algorithm to achieve best accuracy and to study the convergence history as a fundamental characteristic of our method. The PINN is trained until both loss function and the estimated parameters reach a relative plateau. With such a setup, the computational time for case 4, for example, is around 11 hours on a typical machine [with central processing unit (CPU) only] to complete the entire 1M iterations and achieve high accuracy. We note that reasonable accuracy has been achieved within the first 200k iterations. One may further combine Adam and the Limited-memory Broyden–Fletcher–Goldfarb–Shanno (L-BFGS) optimizer (*48*) to achieve similar accuracy within much less computational time (around 30 min; see section S11 and table S1 for detailed results). Recently, parallel PINNs (*49*) have been proposed to accelerate the learning process of PINNs by using multiple CPUs and graphics processing units (GPUs) and introducing parallel algorithms. In addition, other studies have focused on analyzing convergence rate of PINNs and proposing practical techniques for accelerating convergence (*50*–*52*). With the ongoing efforts to improve the original formulation of PINNs, the computational efficiency is expected to be substantially enhanced over time.

We have focused on the prototypical problem as a simple proof of concept, seeking to characterize the internal structures with static loading on outer boundaries. According to Saint-Venant’s principle, under static loading, the inhomogeneous stress and deformation states caused by the internal void/inclusion decay as the distance from the void/inclusion increases. Subsequently, the measurements on outer boundaries essentially provide the PINN with limited amount of information regarding the internal void/inclusion. Modern experimental techniques have adopted dynamic external loading such as ultrasound (*53*) to acquire time-dependent measurements, through which we anticipate that the performance of our method will benefit from more information provided by measurements.

Our method can be applied to a wide range of engineering problems. Defect detection represents a broad class of practical engineering needs in various fields, where identification and characterization of internal structures and defects in materials are essential. Experimental techniques have so far been developed for different materials based on ultrasound (*54*), active thermography (*55*), eddy current (*56*, *57*), optical coherent tomography (*58*), and microwave (*59*). By integrating the respective physical principles in these problems, our approach can potentially be combined with these techniques for dealing with unknown and moving geometries, which extends our method beyond continuum solid mechanics. Notably, one may need to carefully consider the applicability of governing PDEs for practical problems. For instance, continuum solid mechanics does not take into consideration the length scale of microstructures of materials, so that continuum mechanics is accurate only when the key dimensions in the problem (e.g., void size) are much larger than these intrinsic length scales of materials. Our method can also be used for structure design/optimization problems, where typically a mechanical structure is designed with optimized stiffness within volume constraints. For these problems, PINNs can incorporate the design target as a loss term, aspects of which have been preliminarily explored in (*60*).

## MATERIALS AND METHODS

### PINNs for continuum solid mechanics

We introduce the detailed formulation of PINNs for inverse problems in continuum solid mechanics. Here, we focus on the PINN for hyperelasticity (specifically, incompressible Neo-Hookean material) as most of our computational examples adopt this material model (see section S2 for the mechanics of hyperelastic materials). To better clarify the quantitative formulation, here, we denote all the material and geometric parameters of interest as **θ**_mat_ and **θ**_geo_, respectively. For incompressible Neo-Hookean materials, the only material parameter is the shear modulus μ so that **θ**_mat_ = μ. The unknown part of **θ** = (**θ**_mat_, **θ**_geo_) in the inverse problem is denoted as **θ**_unk_.

As summarized in Results, the workflow of PINNs comprises four steps. First, we apply a NN to approximate the primary solution fields (top left panel in Fig. 2B) in domain Ω(**θ**_geo_), including the displacement field $\tilde{u}(X;\lambda )$ and the pressure field $\tilde{p}(X;\lambda )$, where **λ** represents trainable parameters of the NN, **X** = (*X*_1_, *X*_2_) is the in-plane coordinates in the reference/undeformed configuration, and the quantities with tilde represent the approximation from the NN. For incompressible materials, we need the hydrostatic pressure field *p* as a Lagrange multiplier accompanying the displacement field **u** to uniquely determine the stress field.

Second, we integrate mechanical laws into the PINN architecture (top right panel in Fig. 2B) by deriving relevant mechanical quantities of interest from the NN outputs. During this calculation process, partial derivatives are handled by automatic differentiation. The deformation gradient $\tilde{F}(X;\lambda )$ and the first Piola-Kirchhoff stress $\tilde{P}(X;\lambda ,\mu )$ are calculated by

$$\tilde{F}(X;\lambda )=I+\frac{\partial \tilde{u}}{\partial X}(X;\lambda )$$ (4)

$$\tilde{P}(X;\lambda ,\mu )=-\tilde{p}(X;\lambda ){\tilde{F}}^{-T}(X;\lambda )+\mu \tilde{F}(X;\lambda )$$ (5)

where **I** is the identity tensor, Eq. 4 is kinematics, and Eq. 5 is the constitutive relation for incompressible Neo-Hookean materials. The residuals of the equilibrium PDE and the incompressibility condition at **X** are expressed by

$${\tilde{r}}_{\text{PDE}}(X;\lambda ,\mu )=\text{Div}\;\tilde{P}(X;\lambda ,\mu ),\;\;\;X\in \Omega (\theta_{\text{geo}})$$ (6)

$${\tilde{r}}_{\text{inc}}(X;\lambda )=\text{det}(\tilde{F}(X;\lambda ))-1,\;\;\;X\in \Omega (\theta_{\text{geo}})$$ (7)

The residuals of Dirichlet/displacement and Neumann/traction BCs at **X** are

$${\tilde{r}}_{D}(X;\lambda )=\tilde{u}(X;\lambda )-\bar{u}(X),\;\;\;X\in \partial \Omega_{D}(\theta_{\text{geo}})$$ (8)

$${\tilde{r}}_{N}(X;\lambda ,\mu )=\tilde{P}(X;\lambda ,\mu )N(X)-\bar{T}(X),\;\;\;X\in \partial \Omega_{N}(\theta_{\text{geo}})$$ (9)

where **N** is the outward unit normal vector on the boundary, and $\bar{u}$ and $\bar{T}$ are the specified displacement and traction on the boundary, respectively. ∂Ω_D_(**θ**_geo_) and ∂Ω_N_(**θ**_geo_) refer to the domains for Dirichlet/displacement and Neumann/traction BCs, respectively. For inverse problems, we have displacement data ${\left\{u^{*(i)}\right\}}_{i=1}^{N_{u}}$ at ${\left\{X_{u}^{\left(i\right)}\right\}}_{i=1}^{N_{u}}$. The residual of the *i*th displacement observation is

$${\tilde{r}}_{u}^{\left(i\right)}(\lambda )=\tilde{u}(X_{u}^{\left(i\right)};\lambda )-u^{*(i)}$$ (10)

Third, we formulate the loss function according to the foregoing residuals from mechanics and data (bottom right panel in Fig. 2B). To define the loss terms corresponding to the problem definition, we place *N*_Ω_, *N*_D_, and *N*_N_ residual points in Ω, on ∂Ω_D_ and ∂Ω_N_, denoted as $X_{\Omega }^{\left(i\right)}$ (*i* ∈ {1,2, …, *N*_Ω_}), $X_{D}^{\left(i\right)}$ (*i* ∈ {1,2, …, *N*_D_}), and $X_{N}^{\left(i\right)}$ (*i* ∈ {1,2, …, *N*_N_}), respectively. Because we parameterize the coordinates of residual points by **θ**_geo_, these residual points are all parameterized by **θ**_geo_. We evaluate the mean squared residuals of the PDEs, the incompressibility condition, Dirichlet and Neumann BCs, and data, respectively. Each loss term is defined by

$$L_{\text{PDE}}(\lambda ,\theta )=\frac{1}{N_{\Omega }}\sum_{i=1}^{N_{\Omega }}{|{\tilde{r}}_{\text{PDE}}(X_{\Omega }^{\left(i\right)}(\theta_{\text{geo}});\lambda ,\mu )|}^{2}$$ (11)

$$L_{\text{inc}}(\lambda ,\theta )=\frac{1}{N_{\Omega }}\sum_{i=1}^{N_{\Omega }}{|{\tilde{r}}_{\text{inc}}(X_{\Omega }^{\left(i\right)}(\theta_{\text{geo}});\lambda )|}^{2}$$ (12)

$$L_{D}(\lambda ,\theta )=\frac{1}{N_{D}}\sum_{i=1}^{N_{D}}{|{\tilde{r}}_{D}(X_{D}^{\left(i\right)}(\theta_{\text{geo}});\lambda )|}^{2}$$ (13)

$$L_{N}(\lambda ,\theta )=\frac{1}{N_{N}}\sum_{i=1}^{N_{N}}{|{\tilde{r}}_{N}(X_{N}^{\left(i\right)}(\theta_{\text{geo}});\lambda ,\mu )|}^{2}$$ (14)

$$L_{u}(\lambda ,\theta )=\frac{1}{N_{u}}\sum_{i=1}^{N_{u}}{|{\tilde{r}}_{u}^{\left(i\right)}(\lambda )|}^{2}$$ (15)

and the loss function is

$$\begin{array}{c} L(\lambda ,\theta )=\alpha_{\text{PDE}}L_{\text{PDE}}(\lambda ,\theta )+\alpha_{\text{inc}}L_{\text{inc}}(\lambda ,\theta )+\alpha_{D}L_{D}(\lambda ,\theta )\\ +\alpha_{N}L_{N}(\lambda ,\theta )+\alpha_{u}L_{u}(\lambda ,\theta ) \end{array}$$ (16)

where α_PDE_, α_inc_, α_D_, α_N_, and α*_u_* are the weights of the loss terms. Note that the two loss terms ℒ_D_ and ℒ_N_ for the two types of BCs are simplified into ℒ_BC_ in Eq. 1 in Results.

Last, we conduct parameter estimation through training/loss minimization (bottom left panel in Fig. 2B). The trainable parameters of the PINN include the trainable parameters of the NN, **λ**, and the unknown parameters of the inverse problem, **θ**_unk_ (⊆ **θ**). Using the notations in this section, this process can be expressed as

$$\hat{\lambda },{\hat{\theta }}_{\text{unk}}=\underset{\lambda ,\theta_{\text{unk}}\subseteq \theta }{\text{argmin}}L(\lambda ,\theta )$$ (17)

With the PINN adjusting **λ** to minimize the loss function, we anticipate that all the mechanical laws will be approximately satisfied, making the NN serve as an approximation to the primary solution fields. Furthermore, the residual of displacement observations in the loss function guides the estimated unknown parameters to evolve toward their respective target values. In this way, the PINN is able to solve inverse problems.

In section S2, we provide additional information regarding the formulation of PINNs. This includes the formulation for forward problems, for linear elasticity and deformation plasticity, and for multiple materials, which is related to case 5 in our main text.

### Pretraining procedure

We find it necessary to pretrain the model before using the model to characterize unknown geometry. If we directly apply the model without pretraining, then the estimated geometric parameters rapidly depart from physically admissible values (e.g., void located outside the matrix) after a few iterations. Inspired by the transfer learning technique, we propose to maintain all the estimated unknown parameters fixed (not trainable) and only update the trainable parameters of the NN **λ** for the first few iterations. During this pretraining process, the PINN essentially solves a forward problem, seeking to roughly capture the qualitative pattern of the displacement field and the stress field. After this pretraining process, we initiate the parameter estimation process by making both **λ** and **θ** trainable. Such a pretraining procedure induces **λ** to converge to the desired local minimum, hence serving as a good initialization for the geometry identification problem. For our prototypical problem, technically, the PINN needs to be pretrained until there emerges a qualitative pattern indicating the existence of a stress concentration around the void or the soft inclusion.

## Supplementary Materials

### This PDF file includes:

Sections S1 to S11 Figs. S1 to S9 Table S1 References
