# Supplementary material for: Analyses of internal structures and defects in materials using physics-informed neural networks
Source: Sci Adv. 2022 Feb 16;8(7):eabk0644. doi: 10.1126/sciadv.abk0644 (PMC8849303; doi:10.1126/sciadv.abk0644)
Supplement: Supplementary file 1 — Sections S1 to S11 Figs. S1 to S9 Table S1 References [file sciadv.abk0644_sm.pdf]

Science Advances
AAAS

Supplementary Materials for

Analyses of internal structures and defects in materials using physics-informed neural networks

Enrui Zhang, Ming Dao*, George Em Karniadakis*, Subra Suresh*

*Corresponding author. Email: mingdao@mit.edu (M.D.); george_karniadakis@brown.edu (G.E.K.); ssuresh@ntu.edu.sg (S.S.)

Published 16 February 2022, *Sci. Adv.* **8**, eabk0644 (2022)
DOI: 10.1126/sciadv.abk0644

This PDF file includes:

Sections S1 to S11
Figs. S1 to S9
Table S1
References

Section S1. A Brief Review on the Inverse Algorithms for Solid Mechanics

To determine how to update the estimations of unknown parameters, the most widely-used family of methods is based on the calculation of gradients, where the differentiable loss function provides guidance on the increments of estimations. For geometry identification problems, however, the geometry is not naturally differentiable. To address this issue, the shape/topological gradient is proposed by some researchers (*30, 35, 37, 61*), with some studies combined with the level set method (*28*). With the gradient with respect to geometry calculated, the remaining issue is to apply an optimization method to minimize the loss function. Examples of commonly applied optimization algorithms include the L-BFGS algorithm (*36,48,62*), a quasi-Newton iterative method for nonlinear optimization, and the Levenberg-Marquardt method (*33*), a nonlinear least square fitting algorithm. In some other studies, non-gradient methods such as evolutionary algorithms (*32, 34*), have also been applied for updating the geometry.

Section S2. Additional Information on the Formulation of PINNs

Mechanics of Incompressible Hyperelastic Materials. Here we summarize the mechanics of incompressible hyperelastic materials. The reference (undeformed) and current (deformed) configurations of the solid are described by the vectors $\mathbf{X}$ and $\mathbf{x}$, respectively. Then, we may define the displacement vector $\mathbf{u}(\mathbf{X}) = \mathbf{x}(\mathbf{X}) - \mathbf{X}$, the deformation gradient tensor $\mathbf{F}(\mathbf{X}) = \partial\mathbf{x}/\partial\mathbf{X} = \mathbf{I} + \partial\mathbf{u}/\partial\mathbf{X}$ (with the F_{iJ} component being $\partial x_i/\partial X_J$, $i \in \{1, 2, 3\}$, $J \in \{1, 2, 3\}$; $\mathbf{I}$ is the identity tensor), and the right Cauchy-Green tensor $\mathbf{C} = \mathbf{F}^{\mathrm{T}}\mathbf{F}$.

For isotropic, incompressible materials, the three invariants of $\mathbf{C}$ are $I_1 = \mathrm{tr}(\mathbf{C})$, $I_2 = [\mathrm{tr}(\mathbf{C})^2 - \mathrm{tr}(\mathbf{C}^2)]/2$, and $I_3 = \det(\mathbf{C})(= 1$ because of incompressibility). The strain energy density can be expressed as $W(I_1, I_2; \boldsymbol{\theta}_{\text{mat}})$. Specifically, for incompressible Neo-Hookean materials, it takes the form $W(I_1; \mu) = \mu(I_1 - 3)/2$, where the shear modulus μ is the only material parameter ($\boldsymbol{\theta}_{\text{mat}} = \mu$).

The first Piola-Kirchhoff (PK) stress for incompressible materials is expressed as

$$\mathbf{P} = -p\mathbf{F}^{-\mathrm{T}} + 2\mathbf{F}\frac{\partial W}{\partial \mathbf{C}}, \tag{S1}$$

where p is the hydraulic pressure serving as the Lagrange multiplier for incompressible materials to uniquely determine the stress field. For incompressible Neo-Hookean materials,

$$\mathbf{P} = -p\mathbf{F}^{-\mathrm{T}} + \mu\mathbf{F}. \tag{S2}$$

Given the first PK stress tensor $\mathbf{P}$, the equilibrium equation without body force is

$$\mathrm{Div}\ \mathbf{P} = 0, \tag{S3}$$

where the corresponding component form is $\partial P_{iJ}/\partial X_J = 0$ ($i \in \{1, 2, 3\}$; the Einstein summation convention is applied for J).

Now we define the computational domain and boundary conditions. In our current work, we parameterize the domain by $\boldsymbol{\theta}_{\mathrm{geo}}$. Hence, the domain of the PDEs follows as:

$$\mathbf{X} \in \Omega(\boldsymbol{\theta}_{\mathrm{geo}}) \subset \mathbb{R}^d, \tag{S4}$$

where Ω is the parameterized domain of the reference configuration of the problem, and d is the dimension of the problem ($d = 2$ in our cases). The displacement/Dirichlet and traction/Neumann boundary conditions can be expressed as:

$$\mathbf{u} = \overline{\mathbf{u}}, \mathbf{X} \in \partial\Omega_{\mathrm{D}}(\boldsymbol{\theta}_{\mathrm{geo}}), \tag{S5}$$

$$\mathbf{P}\mathbf{N} = \overline{\mathbf{T}}, \mathbf{X} \in \partial\Omega_{\mathrm{N}}(\boldsymbol{\theta}_{\mathrm{geo}}), \tag{S6}$$

where $\mathbf{N}$ is the outward unit normal vector in the reference configuration, and $\partial\Omega_{\mathrm{D}}(\boldsymbol{\theta}_{\mathrm{geo}})$ and $\partial\Omega_{\mathrm{N}}(\boldsymbol{\theta}_{\mathrm{geo}})$ are the displacement and traction boundaries, respectively. The right-hand-side terms $\overline{\mathbf{u}}$ and $\overline{\mathbf{T}}$ are the specified values of displacement and traction at the boundary, respectively. The component form corresponding to Eq. S6 is $P_{iJ}N_J = \overline{T}_i$.

In the case where there is more than one material, the continuity of displacement and traction along the interface $\Gamma_{\text{int}}(\boldsymbol{\theta}_{\text{geo}})$ (between material 1 and 2) must also be satisfied:

$$\mathbf{u}^{(1)} = \mathbf{u}^{(2)}, \mathbf{X} \in \Gamma_{\text{int}}(\boldsymbol{\theta}_{\text{geo}}), \tag{S7}$$

$$\mathbf{P}^{(1)}\mathbf{N} = \mathbf{P}^{(2)}\mathbf{N}, \mathbf{X} \in \Gamma_{\text{int}}(\boldsymbol{\theta}_{\text{geo}}), \tag{S8}$$

where the superscripts refer to the materials 1 and 2, respectively, and $\mathbf{N}$ is the unit normal vector of the interface Γ_{int} in the reference configuration.

PINNs for Linear Elasticity and Deformation Plasticity We summarize the differences in the architecture of PINNs for compressible linear elasticity (Fig. 2A) and deformation plasticity (Fig. 2C) compared to incompressible hyperelasticity (Fig. 2B). For these two constitutive relations, the solid undergoes infinitesimal deformation, so that one does not need to distinguish reference (undeformed) configuration (X_1, X_2) and deformed configuration (x_1, x_2). In this section, the coordinates are written as (x_1, x_2) to keep consistent with the conventional notations in solid mechanics community. Due to the compressibility, the hydrostatic pressure p is no longer an independent state variable, so that p is not needed as a primary output of the NN.

To build the PINN for linear elasticity, kinematics in Eq. 4 in the main text is replaced by the definition of the infinitesimal strain tensor $\varepsilon_{ij} = \partial u_i / \partial x_j$, where $\mathbf{u} = (u_1, u_2)$ is the displacement. Stress-deformation relationship in Eq. 5 is replaced by the stress-strain relationship

$$\sigma_{ij} = \lambda \varepsilon_{kk} \delta_{ij} + 2\mu \varepsilon_{ij}, \tag{S9}$$

where λ and μ are Lamé constants. The equilibrium equation similar to Eq. 6 is $\partial \sigma_{ij} / \partial x_j = 0$. Note that the loss function for linear elasticity does not require the term for incompressibility (see Eq. 1 and Eq. 16).

The PINN for the power-law deformation plasticity can be constructed based on the PINN

for linear elasticity. In this case, the nonlinear stress-strain relation is expressed as

$$E\boldsymbol{\varepsilon} = \boldsymbol{\sigma} + \frac{3}{2}\alpha\left(\frac{\sigma_{\mathrm{e}}}{\sigma_{\mathrm{Y}}}\right)^{n-1}\boldsymbol{s}, \tag{S10}$$

where E (Young's modulus), α, σ_{Y} (yield stress), and n (hardening exponent) are material parameters. $\boldsymbol{s}$ is the deviatoric stress tensor ($s_{ij} = \sigma_{ij} - \frac{1}{3}\sigma_{kk}\delta_{ij}$), and σ_{e} ($= \sqrt{\frac{3}{2}s_{ij}s_{ij}}$) is the von Mises stress.

According to Eq. S10, stress cannot be explicitly expressed by strain for power-law deformation plasticity, unlike linear elasticity (Fig. 2A) and hyperelasticity (Fig. 2B). Therefore, the integration of constitutive relation through the analytical expression of stress tensor no longer works for deformation plasticity. As an alternative approach, we include the stress field $\boldsymbol{\sigma} = (\sigma_{11}, \sigma_{12}, \sigma_{22})$ as primary solution field of the neural network in addition to the displacement field (u_1, u_2) (see Fig. 2C). After calculating the strain field, we bridge the strain and stress fields with an additional loss term according to Eq. S10. In this way, we integrate constitutive relation as an additional penalty term in the loss function for deformation plasticity.

Multiple materials. In the case of inverse problems for multiple materials as in case 5 in the main paper, we apply two independent NNs to approximate the displacement and pressure fields of the matrix and inclusion materials, respectively. Such approximation can be expressed by

$$\text{Matrix: } (\widetilde{\mathbf{u}}(\mathbf{X};\boldsymbol{\lambda}_{\mathrm{m}}), \widetilde{p}(\mathbf{X};\boldsymbol{\lambda}_{\mathrm{m}})), \mathbf{X} \in \Omega_{\mathrm{m}}(\boldsymbol{\theta}_{\mathrm{geo}}) \tag{S11}$$

$$\text{Inclusion: } (\widetilde{\mathbf{u}}(\mathbf{X};\boldsymbol{\lambda}_{\mathrm{i}}), \widetilde{p}(\mathbf{X};\boldsymbol{\lambda}_{\mathrm{i}})), \mathbf{X} \in \Omega_{\mathrm{i}}(\boldsymbol{\theta}_{\mathrm{geo}}) \tag{S12}$$

In addition to the loss terms for each material, we need an additional loss term $\mathcal{L}_{\mathrm{int}}$ to force the continuity of displacement and traction on the interface $\Gamma_{\mathrm{int}}(\boldsymbol{\theta}_{\mathrm{geo}})$ of two materials. To do this, we first write the residuals on a single point for the two conditions of continuity. The residual of displacement continuity is

$$\widetilde{\mathbf{r}}_{\mathrm{Dint}}(\mathbf{X};\boldsymbol{\lambda}_{\mathrm{m}},\boldsymbol{\lambda}_{\mathrm{i}}) = \widetilde{\mathbf{u}}(\mathbf{X};\boldsymbol{\lambda}_{\mathrm{m}}) - \widetilde{\mathbf{u}}(\mathbf{X};\boldsymbol{\lambda}_{\mathrm{i}}), \mathbf{X} \in \Gamma_{\mathrm{int}}(\boldsymbol{\theta}_{\mathrm{geo}}), \tag{S13}$$

and the residual of stress continuity is

$$\widetilde{\mathbf{r}}_{\text{Nint}}(\mathbf{X};\boldsymbol{\lambda}_{\text{m}},\boldsymbol{\lambda}_{\text{i}},\mu_{\text{m}},\mu_{\text{i}}) = \widetilde{\mathbf{P}}(\mathbf{X};\boldsymbol{\lambda}_{\text{m}},\mu_{\text{m}})\mathbf{N}(\mathbf{X}) - \widetilde{\mathbf{P}}(\mathbf{X};\boldsymbol{\lambda}_{\text{i}},\mu_{\text{i}})\mathbf{N}(\mathbf{X}), \mathbf{X} \in \Gamma_{\text{int}}(\boldsymbol{\theta}_{\text{geo}}). \quad \text{(S14)}$$

We place residual points $\mathbf{X}_{\Gamma}^{(i)}(\boldsymbol{\theta}_{\text{geo}})$ ($i \in \{1, 2, ..., N_{\Gamma}\}$) on $\Gamma(\boldsymbol{\theta}_{\text{geo}})$, and construct the loss component on the interface of two materials by taking a weighted sum as

$$\mathcal{L}_{\text{int}}(\boldsymbol{\lambda}_{\text{m}},\boldsymbol{\lambda}_{\text{i}},\boldsymbol{\theta}) = \alpha_{\text{Dint}}\mathcal{L}_{\text{Dint}}(\boldsymbol{\lambda}_{\text{m}},\boldsymbol{\lambda}_{\text{i}},\boldsymbol{\theta}) + \alpha_{\text{Nint}}\mathcal{L}_{\text{Nint}}(\boldsymbol{\lambda}_{\text{m}},\boldsymbol{\lambda}_{\text{i}},\boldsymbol{\theta}), \quad \text{(S15)}$$

where

$$\mathcal{L}_{\text{Dint}}(\boldsymbol{\lambda}_{\text{m}},\boldsymbol{\lambda}_{\text{i}},\boldsymbol{\theta}) = \frac{1}{N_{\Gamma}}\sum_{i=1}^{N_{\Gamma}}\left|\widetilde{\mathbf{r}}_{\text{Dint}}\left(\mathbf{X}_{\Gamma}^{(i)}(\boldsymbol{\theta}_{\text{geo}});\boldsymbol{\lambda}_{\text{m}},\boldsymbol{\lambda}_{\text{i}}\right)\right|^2 \quad \text{(S16)}$$

$$\mathcal{L}_{\text{Nint}}(\boldsymbol{\lambda}_{\text{m}},\boldsymbol{\lambda}_{\text{i}},\boldsymbol{\theta}) = \frac{1}{N_{\Gamma}}\sum_{i=1}^{N_{\Gamma}}\left|\widetilde{\mathbf{r}}_{\text{Nint}}\left(\mathbf{X}_{\Gamma}^{(i)}(\boldsymbol{\theta}_{\text{geo}});\boldsymbol{\lambda}_{\text{m}},\boldsymbol{\lambda}_{\text{i}},\mu_{\text{m}},\mu_{\text{i}}\right)\right|^2. \quad \text{(S17)}$$

Hence, the loss function in the case of multiple materials is the summation of the loss function for each single material and the additional loss $\mathcal{L}_{\text{int}}$ for the material interface.

PINNs for forward problems. For forward problems with no unknown parameter ($\boldsymbol{\theta}_{\text{unk}} = \emptyset$, hence $\boldsymbol{\theta} = \emptyset$ for convenience), we do not have measurement data. For incompressible materials, for example, the loss function can be written as a weighted sum of all the four loss terms that correspond to PDEs, the incompressibility condition, displacement boundary conditions, and traction boundary conditions, respectively:

$$\mathcal{L}_{\text{for}}(\boldsymbol{\lambda}) = \alpha_{\text{PDE}}\mathcal{L}_{\text{PDE}}(\boldsymbol{\lambda}) + \alpha_{\text{inc}}\mathcal{L}_{\text{inc}}(\boldsymbol{\lambda}) + \alpha_{\text{D}}\mathcal{L}_{\text{D}}(\boldsymbol{\lambda}) + \alpha_{\text{N}}\mathcal{L}_{\text{N}}(\boldsymbol{\lambda}). \quad \text{(S18)}$$

Each loss term is defined by

$$\mathcal{L}_{\text{PDE}}(\boldsymbol{\lambda}) = \frac{1}{N_\Omega} \sum_{i=1}^{N_\Omega} \left| \widetilde{\mathbf{r}}_{\text{PDE}}\left(\mathbf{X}_\Omega^{(i)}; \boldsymbol{\lambda}\right) \right|^2 \tag{S19}$$

$$\mathcal{L}_{\text{inc}}(\boldsymbol{\lambda}) = \frac{1}{N_\Omega} \sum_{i=1}^{N_\Omega} \left| \widetilde{r}_{\text{inc}}\left(\mathbf{X}_\Omega^{(i)}; \boldsymbol{\lambda}\right) \right|^2 \tag{S20}$$

$$\mathcal{L}_{\text{D}}(\boldsymbol{\lambda}) = \frac{1}{N_\text{D}} \sum_{i=1}^{N_\text{D}} \left| \widetilde{\mathbf{r}}_{\text{D}}\left(\mathbf{X}_D^{(i)}; \boldsymbol{\lambda}\right) \right|^2 \tag{S21}$$

$$\mathcal{L}_{\text{N}}(\boldsymbol{\lambda}) = \frac{1}{N_\text{N}} \sum_{i=1}^{N_\text{N}} \left| \widetilde{\mathbf{r}}_{\text{N}}\left(\mathbf{X}_\text{N}^{(i)}; \boldsymbol{\lambda}\right) \right|^2. \tag{S22}$$

The solution of forward problems using PINNs can be expressed as:

$$\hat{\boldsymbol{\lambda}} = \underset{\boldsymbol{\lambda}}{\operatorname{argmin}}\, \mathcal{L}_{\text{for}}(\boldsymbol{\lambda}), \tag{S23}$$

The displacement solved by the PINN is $\widetilde{\mathbf{u}}(\mathbf{X}; \hat{\boldsymbol{\lambda}})$ for $\mathbf{X} \in \Omega$.

Section S3. Technical Details

Details of the Prototypical Problem. For case 0, the Young's modulus of the matrix is $E = 1.0$, the Poisson's ratio is $\nu = 0.3$, and the loading is $P_0 = 0.003$. For cases 1, 2, 4 and 5, the shear modulus of the matrix (incompressible Neo-Hookean material) is $\mu_\text{i} = 0.333$, and the external load is $P_0 = 0.3$. For case 3, the external load is $P_0 = 0.002$, and the material parameters include $E = 1.0$, $\alpha = 0.1$, $n = 10$ and $\sigma_\text{Y} = 0.005$. For cases 0, 1, 3, 4, 5, there are $N_u = 10$ measurement points of on each edge of the matrix. For case 2, measurement points are inside the solid, with 10 of them uniformly placed on the line $X_1 = -0.15$, $X_1 = 0.15$, $X_2 = -0.45$, $X_2 = 0.45$, respectively. For the modified case 5 with additional measurements, the five additional measurement points are located at $(-0.3, 0.05)$, $(-0.3, 0.10)$, $(-0.3, 0.15)$ in the matrix and $(-0.05, 0.10)$, $(0.15, 0.10)$ in the inclusion.

Details of the PINN. We use TensorFlow 1.14 (*43*) to build up our PINN. Each NN in the PINN has 4 hidden layers, each with 30 neurons. For cases 0, 1, 2 and 4, there is one single

NN in the PINN. There are two NNs in the PINN in case 3 (one NN for (u_1, u_2), the other NN for $(\sigma_{11}, \sigma_{22}, \sigma_{12})$) and case 5 (one NN for each material). We adopt the layer-wise adaptive "tanh" function (*63*) as the activation function for the NNs. Among the trainable parameters of the NN, weights are initialized with Xavier initialization (*64*), biases are initialized as zeros, and the variable of adaptive activation are initialized as ones. We use the Adam optimizer (*47*) to train the network. The learning rate is 0.001 for all cases except cases 2 and 3.

For case 2, the unknown geometric parameters directly defined in the code are the location of the center of the slit $(X_1^{(c)}, X_2^{(c)})$, half length of the slit L, and tilting angle of the slit Γ, which are post-processed to be the coordinates of the locations of the centers of the slit tips $(X_1^{(1)} = X_1^{(c)} - L\sin\Gamma$, $X_2^{(1)} = X_2^{(c)} + L\cos\Gamma$, $X_1^{(2)} = X_1^{(c)} + L\sin\Gamma$, $X_2^{(2)} = X_2^{(c)} - L\cos\Gamma)$. The learning rate is 0.001 for Γ and 0.0002 for $(X_1^{(c)}, X_2^{(c)}, L)$. For case 3, the learning rate is 0.00005.

For cases 0, 1, 3 and 5, the assignments of residual points are similar. We assign 3200 internal points for the PDE and incompressibility (800 points for each of the four sub-regions; 40 along the circumferential direction and 20 along the radial direction). For case 5 only, 400 residual points are assigned in the inclusion material. We place 160 residual points on the outer boundary for enforcing traction boundary conditions. Another 160 residual points are placed on the inner boundary of the matrix, which enforce the traction-free boundary conditions for cases 1 and 3 and the interface conditions for case 5. The points of displacement measurements are uniformly placed on the outer boundary of the matrix.

For case 2, the smallest length scale is determined by the W, which poses a limitation for the interval of residual points. To accurately resolve the displacement field, especially around the slit tip where the displacement changes drastically and the stress concentration exists, we need a large density of residual points in the computational domain. For this case, we have 49000 internal points, 700 points on the outer boundary, and 700 points on the inner boundary.

For case 4, we divide the square region into two parts, each with one void. For each part, residual points are assigned in a similar way to case 1.

For all the cases, we choose the weights of the loss terms in Eq. 16 to be $\alpha_{\text{PDE}} = \alpha_{\text{inc}} = \alpha_{\text{D}} = \alpha_u = 1$ when applicable. The weight for traction boundary conditions α_{N} is also set to be 1, but we evaluate the this part separately for each of the five boundaries (left, right, top, bottom, inner), leading to five loss terms related to traction boundaries, each with weight 1, in the total loss. In case 2, the calculation of the mean squared error of the PDE loss term $\mathcal{L}_{\text{PDE}}(\boldsymbol{\lambda}, \boldsymbol{\theta})$ is further weighed inversely by the local density of residual points, so that different spatial regions contribute equally to the loss term of PDEs.

For cases 0 and 3, the small values of loading (roughly $\mathcal{O}(10^{-3})$) cause difficulties in training the PINN. To mitigate this issue, we scale up the external loading (and $\sigma_{\text{Y}} = 0.005$ in case 3) 100 times and feed the scaled loading into the PINN. According to Eqs. S9 and S10, such scaling only results in 100 times the original displacement (and hence strain) and does not essentially changes the characteristics of the solution. After obtaining the solution from the PINN, we scale the displacement/strain solution back (0.01 times) to its original value.

Details of the FEM. We use Abaqus as the FEM solver to generate the displacement data $\{\mathbf{u}^{*(i)}\}_{i=1}^{N_u}$ and the entire displacement field, given the reference values of unknown parameters $\boldsymbol{\theta}^*_{\text{unk}}$. The linear density of meshes is 120 per unit length, which is sufficiently dense so that the FEM/Abaqus solution is accurate enough to serve as the reference solution. Plane strain quadratic elements with hybrid formation (CPE8H elements) are applied for hyperelasticity.

Section S4. Additional Results for Cases in the Main Text

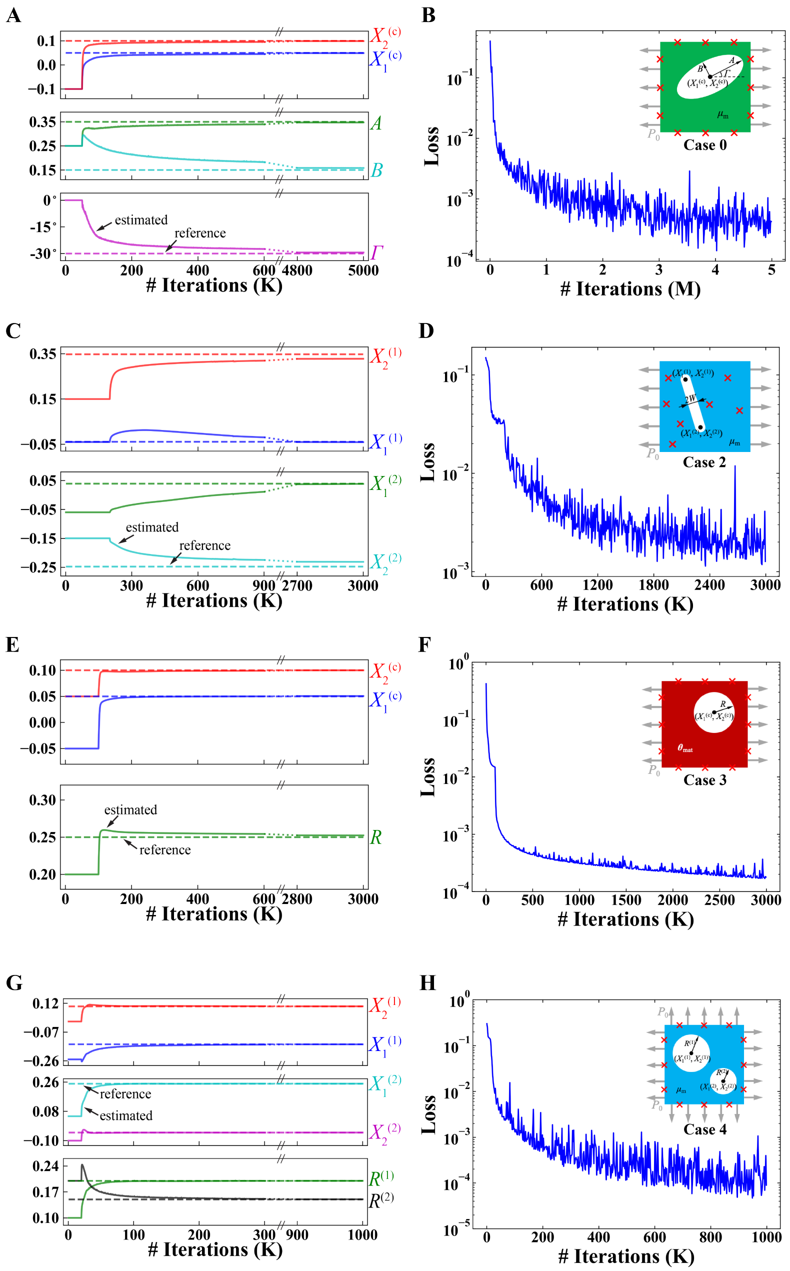

Fig. S1. Evolution of estimated unknown parameters and loss function in cases 0, 2, 3 and 4. See Fig. 2 for the definitions of the cases. (**A**, **C**, **E**, **G**) The dashed lines and solid lines represent the reference value and estimated value of unknown variables. Unknown parameters are not trainable in the pre-training process during the first 50K (case 0), 200K (case 2), 100K (case 3), and 20K (for case 4) iterations, respectively. (**B**, **D**, **F**, **H**) Loss function during the training process. See Fig. 7 in the main text for the results for cases 1 and 5.

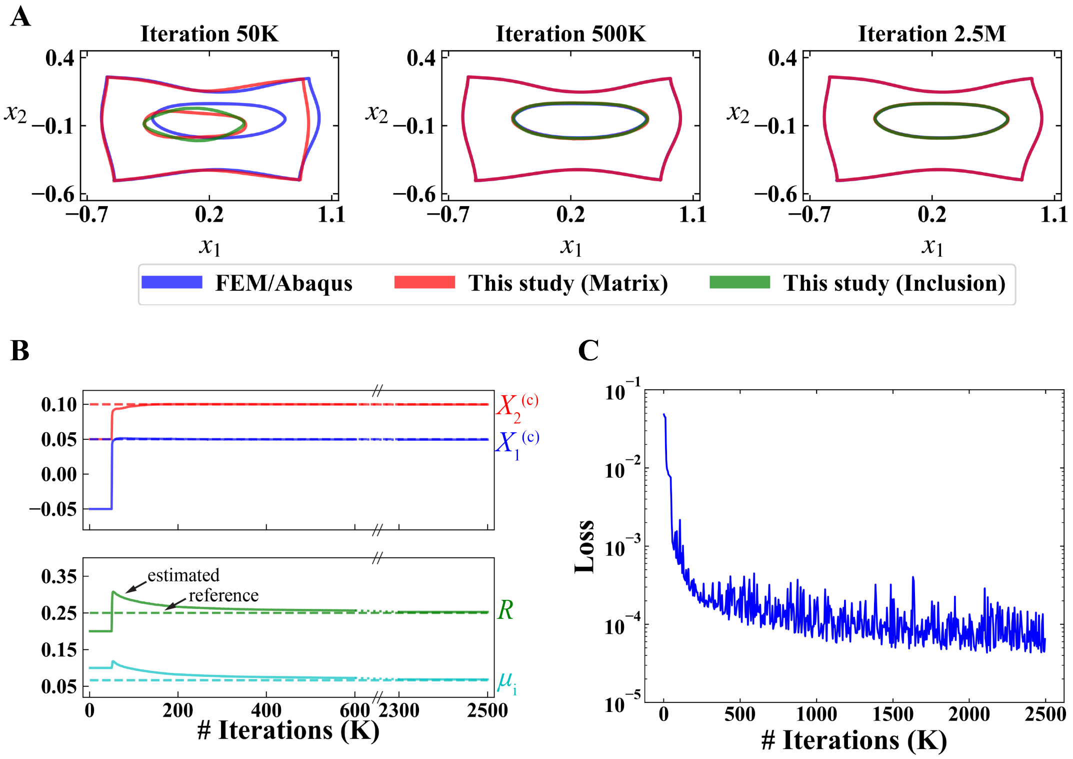

Fig. S2. Results of case 5 with additional internal measurement points. (**A**) Comparison of the visual outlines (including the material interface) of the deformed configurations between FEM/Abaqus (blue) and PINN (red for the matrix; green for the inclusion) results after 50K, 500K, and 2.5M iterations. (**B**) Evolution of the estimated unknown parameters $(X_1^{(c)}, X_2^{(c)}, R, \mu_i)$ during the training process. The dashed lines and solid lines are reference values and estimated values, respectively. (**C**) The loss function during the training process.

Section S5. Forward Problem on One Centered Circular Void

In this section, we study on a forward problem to verify the ability of PINNs in solving boundary value problems of hyperelastic solids (see Section S2 for details of the formulation of PINNs for forward problems). For forward problems, we have $\boldsymbol{\theta}_{\text{unk}} = \emptyset$ (hence $\boldsymbol{\theta} = \emptyset$ for convenience), and we do not have any observation on the displacement field. The setup is a simplified, forward version of case 1: the matrix includes a circular void located at the center $(0.0, 0.0)$ with radius 0.1; all the other setup details are the same as the foregoing description for case 1. The target is to solve the displacement field under the specified loading condition.

The results are shown in Fig. S3. We trained the PINN over 1M iterations in total. Fig. S3A shows the comparison of deformed configurations of the solid between the FEM solver and the PINN solver after 20K, 200K and 1M iterations, with the absolute L^2 error of the displacement field marked on each subfigure. For clarity of presentation, this figure shows the outer and inner boundaries of the specimen visualized from the FEM and PINN analyses. Although we trained the PINN for 1M iterations in total, the visual pattern of the PINN result almost fully overlaps with the pattern of the FEM result after 200K iterations. The L^2 error finally decreases to as small as $\mathcal{O}(10^{-4})$, compared to the typical displacement of $\mathcal{O}(10^{-1})$. Fig. S3B shows the distribution of the true stress component σ_{11} along the central line of the solid ($X_1 = 0$) after 20K, 200K and 1M iterations. After training over 20K iterations, the stress pattern of the PINN result is already qualitatively similar to that of the FEM result. The remaining difference is gradually diminishing through the following training iterations. Fig. S3C shows the value of the loss function throughout the training process. The total loss is decreased to $\mathcal{O}(10^{-5})$ after 1M iterations. According to all the results presented in Fig. S3, the PINN has accurately solved this forward boundary value problem of hyperelastic solids. This example of a forward problem serves as a basis for the inverse problem where the introduction of trainable unknown parameters slightly increases the complexity of the problem. In Section S6, we present a parametric study based on this case.

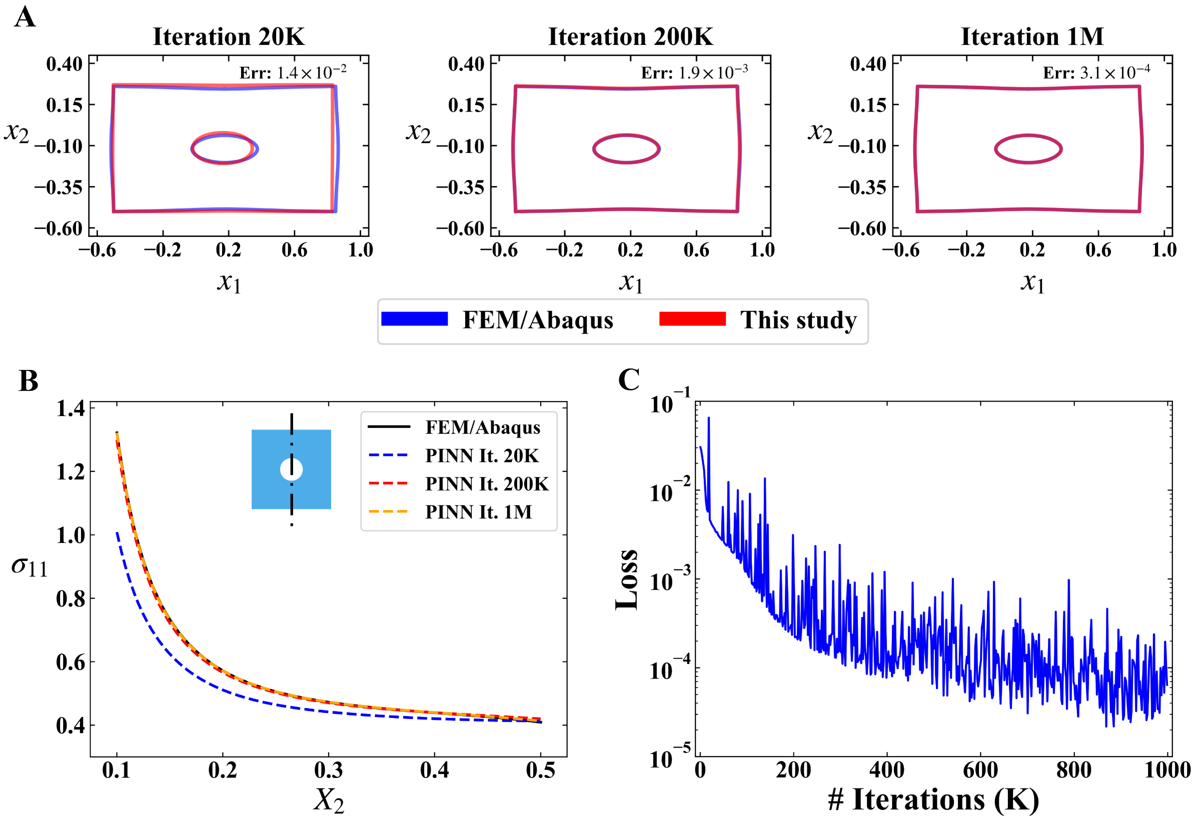

Fig. S3. Results of the forward problem on one centered circular void. (**A**) Comparison of the visual outlines of deformed configurations between FEM/Abaqus (blue) and PINN (red) results after 20K, 200K, and 1M iterations. The absolute L^2 error of the displacement field between the two methods is marked in each subfigure. (**B**) Stress component σ_{11} along the vertical axis of symmetry ($X_1 = 0$) after 20K, 200K, and 1M iterations (dashed lines), with the FEM/Abaqus result as a comparison (solid line). (**C**) The loss function during the training process.

Section S6. Parametric Study of the Forward Problem: Influence of NN Architecture, Density of Residual Points, and Loss Weights

Here we conduct a parametric study on the forward problem in Section S5. We study the influence of hyperparameters on the simulation results to justify choice of hyperparameters in this paper. We consider changing the width and the depth of the NN from the reference architecture 2-30-30-30-30-3 ($d = 4$ hidden layers each with width $w = 30$, called 4-30 for

short and similarly d-w for other architectures) in the main paper to be wider (4-45), narrower (4-15), deeper (6-30), and shallower (2-30). In the forward problem in Section S5 (and also case 1 in main text), we have placed 40×20 residual points in each of the four sub-regions (see Section S3 for details). Here we change the number of residual points into variables: $\rho \times (\rho/2)$ for each sub-region, where ρ is the linear density of residual points. With this definition, the number of residual points on the boundary is proportional to ρ, and the number of residual points inside the solid is proportional to ρ^2. Here we consider changing ρ to range from 10 to 80. For each combination of ρ and d-w, we use the PINN to solve the forward problem. The results of the absolute L^2 error of the displacement field compared to the FEM solution are shown in Fig. S4. We consider the displacement fields after 200K (averaged by taking the geometric mean value after 180K, 190K and 200K iterations, to prevent the influence of fluctuation) and 1M iterations (averaged similarly by 980K, 990K and 1M iterations) . For any architecture, as ρ increases to around 30, the L^2 error does not significantly depend on ρ, indicating that the chosen values of ρ in the main paper are sufficiently large for achieving a high accuracy. From 200K to 1M iterations, the L^2 errors of the shallower (2-30) and the narrower (4-15) architectures do not further decrease significantly, while the errors of the reference (4-30), deeper (6-30), and wider (4-45) architectures continue decreasing significantly. This indicates that the insufficient depth for 2-30 and width for 4-15 limits the approximation ability of the NN and hence restricts the further descent of the error. The reference architecture (4-30) is deep and wide enough for reaching a small L^2 error, because its error is close to the error from the deeper (6-30) and wider (4-45) architectures. This simple parametric study indicates that our choice of the architecture of the NN (4-30) and the linear density of the residual points (40) is suitable for solving our prototypical problem.

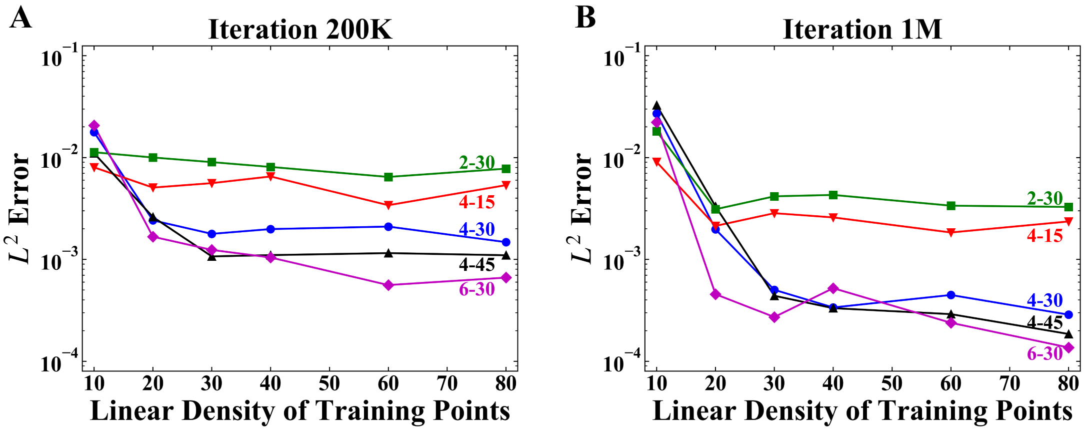

Fig. S4. Parametric study of the forward problem in Section S5 on the NN architecture and density of residual points. We compare the L^2 error of displacement fields obtained by the PINN and the FEM/Abaqus after training over (**A**) 200K and (**B**) 1M iterations. We consider different depths (2, 4, and 6 hidden layers) and widths (15, 30, and 45 neurons for each hidden layer) of the NN, as well as different linear densities of residual points. The results of the network with d hidden layers and w neurons for each hidden layer are marked with "d-w". The linear density of residual points ρ means that the number of residual points is proportional to ρ for the boundary conditions and proportional to ρ^2 for the PDEs. The displayed value of L^2 error is obtained by taking the geometric mean value after 180K, 190K and 200K iterations for (**A**) and 980K, 990K and 1M iterations for (**B**), in order to prevent the fluctuation during the training process.

We also consider changing the weights of the loss terms (α's with various subscripts) and studying their influence on the convergence histories of the loss function and the accuracy of the displacement field. Again, we conduct the study using the forward problem in Section S5. For simplicity, we assume $\alpha_{\text{PDE}} = \alpha_{\text{inc}} = 1$ and $\alpha_{\text{D}} = \alpha_{\text{N}} = \alpha$, where α is a variable. With this assumption, we group the loss weights by whether the corresponding residual points are inside the domain or on the boundary. Note that in Section S5, the choice is $\alpha = 1$. Here we train the PINN with $\alpha = 1, 3, 10$ and all the other setup details the same as Section S5. The results are shown in Fig. S5. Figs. S5A-C show the evolution of the total loss ($\mathcal{L}_{\text{for}}$), the internal loss ($\mathcal{L}_{\text{PDE}} + \mathcal{L}_{\text{inc}}$), and the boundary loss ($\mathcal{L}_{\text{D}} + \mathcal{L}_{\text{N}}$) during the training process for $\alpha = 1, 3, 10$, respectively. As α increases, the boundary loss tends to descend faster, while the

evolution of the internal loss and the total loss does not change significantly. Fig. S5D shows the evolution of the absolute L^2 errors of the displacement fields for $\alpha = 1, 3, 10$ compared to the FEM/Abaqus solution. With a larger α, the displacement field converges faster. On the other hand, in the late stage the training process (after 500K iterations), the three values of L^2 error reach the same plateau, meaning that the value of α does not significantly influence the final accuracy of the displacement field for the forward problem. Since this paper focuses on the fundamental method and the prototypical problem as a proof of concept rather than tuning the hyperparameters to achieve optimal practical efficiency, we simply fix all the weights to be one in all the cases in the main text.

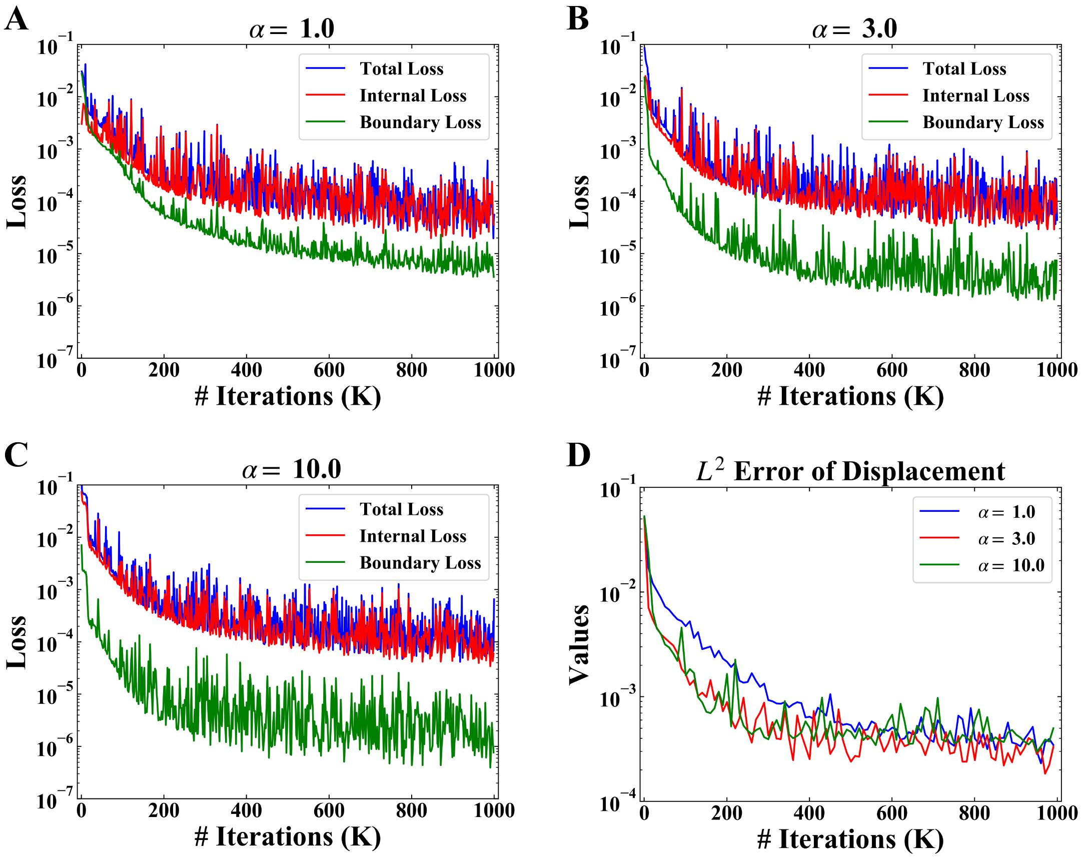

Fig. S5. Parametric study on the weights of loss components. We studied the influence of loss weights on the evolution of the loss function and the accuracy of the displacement using the forward problem in Section S5. We fix the weights of the loss terms inside the PDE domain to be $\alpha_{\text{PDE}} = \alpha_{\text{inc}} = 1$, and set the loss terms on the boundary to be one variable $\alpha_{\text{D}} = \alpha_{\text{N}} = \alpha$. (**A-C**) The evolution of the total loss ($\mathcal{L}_{\text{for}}$), the internal loss ($\mathcal{L}_{\text{PDE}} + \mathcal{L}_{\text{inc}}$), and the boundary loss ($\mathcal{L}_{\text{D}} + \mathcal{L}_{\text{N}}$) during the training process for $\alpha = 1, 3, 10$, respectively. (**D**) The absolute L^2 error of the displacement field by the PINN compared to the FEM/Abaqus result for $\alpha = 1, 3, 10$ during the training process.

Section S7. Parametric Study of Simplified Case 1: Influence of Locations of Measurement Points on the Outer Boundary

In this section, we conduct a parametric study on the influence of locations of measurement points on the estimation results based on case 1. We alter the locations of displacement measurements on the outer boundary, while keeping all the other setup details unchanged. The six

setups of displacement measurements (sensors) are illustrated in Fig. S6. In all the setups, 10 points are placed on the left and right boundaries, respectively. To create a relatively tough condition, there is no measurement is on the top and bottom boundaries. The difference among six setups comes from the specific locations of measurement points. The left and right edges are equally partitioned into 3 segments, with different segments equipped with measurement points in different setups: the bottom segment for setup 1, the middle segment for setup 2, the top segment for setup 3, the bottom and middle segments for setup 4, the middle and top segments for setup 5, and all segments for setup 6 (see Fig. S6A).

The tilting angle Γ is assumed to be the only unknown parameter for the study in this section. The evolution of Γ (normalized by $180°$) in the six setups over 1M training iterations is shown in Fig. S6B. The reference value Γ^* is plotted with dashed line. In setups 3, 5 and 6, the estimated values of Γ gradually approach the reference value, despite the existence of errors which may be caused by the removal of measurement points on the top and bottom boundaries. In setups 1, 2 and 4, the estimated Γ do not approach the target value, indicating that the parameter estimation fails. We further show the deformed configuration of each setup after 870K iterations in Fig. S6C, where the fluctuating values of Γ in setup 2 and 4 are accidentally close to the reference value Γ^*. According to Fig. S6C, setups 3, 5 and 6 infer the displacement field accurately. For setups 2 and 4, although Γ happens to be close to Γ^*, their deformed configurations are significantly different from the FEM results. Consequently, setups 3, 5 and 6 successfully estimate the unknown tilting angle and infer the displacement field, while setups 1, 2 and 4 fail to do so.

For the specific example we consider, a successful characterization of geometry requires that the measurement points are on the top segment, while the middle and bottom segments do not contribute much. This feature may be caused by the fact that the actual location of the void is slightly above the center ($X_2^{(c)*} > 0$). Intuitively, the closer the measurement points are to

the void, the more informative the data is. This conforms with the Saint-Venant's principle in elasticity – if the measurements are located too far from the void, then the non-homogeneity of the displacement field is diminished, so that these measurements do not contain enough information regarding the void geometry. Therefore, solving geometry identification problems with PINNs poses requirements on the location of measurement points. For the current problem, without prior knowledge of the location and mechanical properties of the void, a safe way is to place the measurement points uniformly in the admissible region (e.g., on the outer boundary) to acquire as much information as possible.

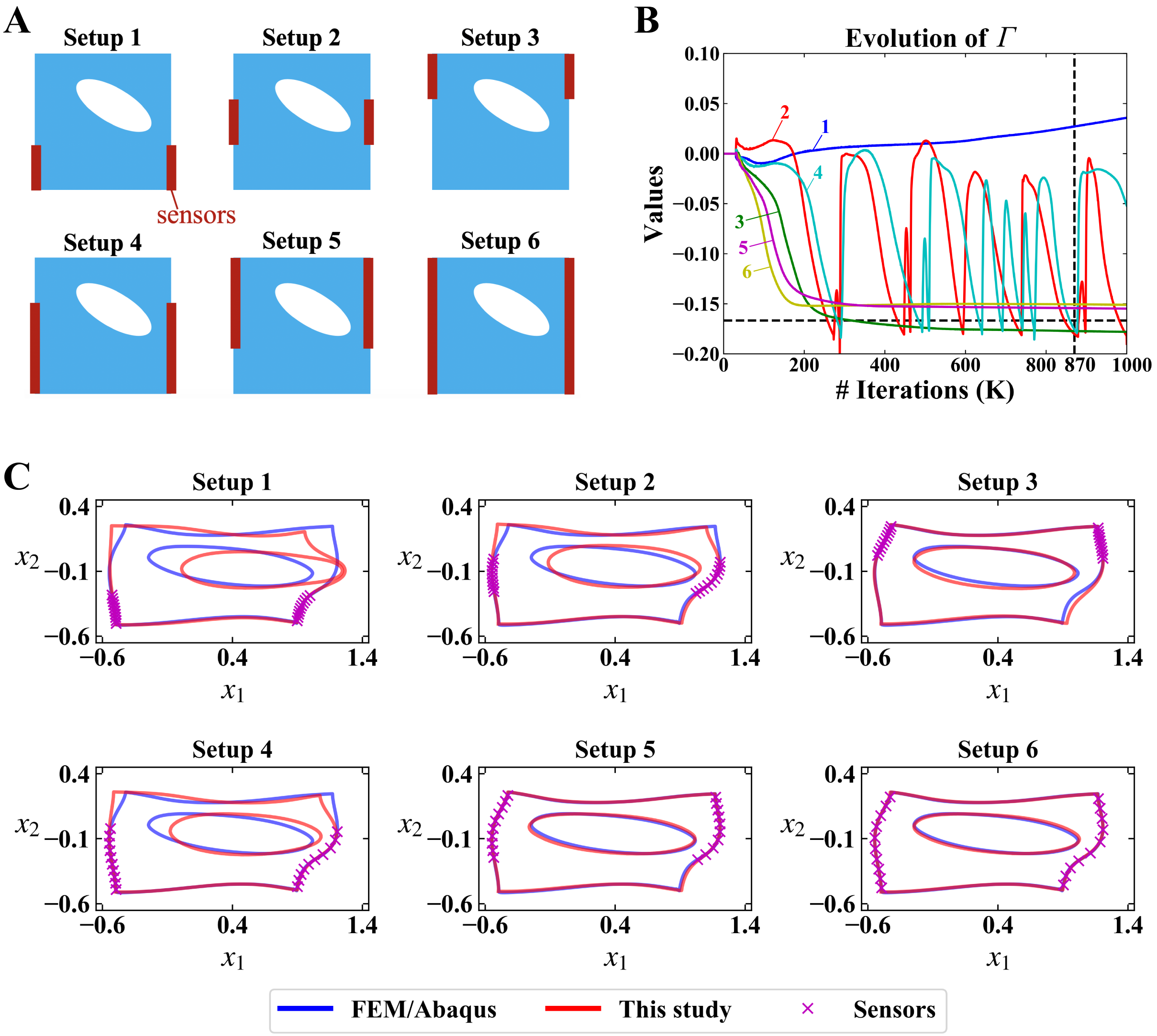

Fig. S6. Parametric study on the locations of measurement points. We show the dependence of parameter estimation results on the locations of measurement points. (**A**) In the 6 different setups, we place 10 points uniformly at different segments of the left and right boundaries, respectively. The left and right edges are equally partitioned into 3 segments, and then measurement points (sensors) are placed on: the bottom segment for setup 1, the middle segment for setup 2, the top segment for setup 3, the bottom and middle segments for setup 4, the middle and top segments for setup 5, and all segments for setup 6. (**B**) Parameter estimation results of the tilting angle Γ (normalized by 180°) in the 6 setups. The curves for different setups are marked with the setup numbers. The horizontal dashed line is the reference value Γ^*. The vertical dashed line marks the location of 870K training iterations. (**C**) The deformation patterns of all setups after 870K iterations.

Section S8. Parametric Study on Simplified Case 1: Influence of the Length Scale of the Void

We in this section study the influence of the length scale of the void on the estimation accuracy. For the purpose of simplicity, we assume that the void is circular and is placed at the center of the matrix, with the only unknown parameter being its radius ($\boldsymbol{\theta}_{\text{unk}} = R$). We set the radius of the void R^* to range from 0.025 to 0.2, and initialize the radius estimation to be $R^0 = 1.5R^*$ for each case. For each R^*, we run the training process until the estimated value converges evidently. Other technical details are the same as case 1 of the main paper.

The results of the relative error of estimated radius are shown in Fig. S7. Note that the side length of the matrix is 1.0. As R^* decreases to around 0.1, the relative error starts to increase beyond 10^{-2}. As the radius decreases to $R^* = 0.05$, the identification error is around 20%, indicating that the method starts to become inaccurate. Consequently, for this specific case where displacement is measured only on the boundary, our approach is valid for voids within around one order of magnitude smaller than the size of the entire domain.

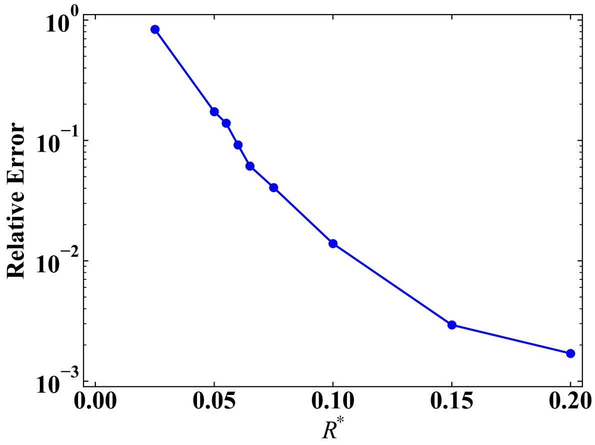

Fig. S7. Parametric study on the length scale of the void. We show the relative error of the estimated void radius for different values of R^* (radius of the void).

Section S9. Parametric Study of Simplified Case 1: Influence of the Location of the Void

A natural question following Section S8 is how the location of the void influences the estimation accuracy. Here we consider another simplified setup of Case 1. We suppose that only $(X_1^{(\mathrm{c})}, X_2^{(\mathrm{c})}, R)$ are unknown to the PINN. We fix $R^* = 0.1$ and change the location of the void by choosing $X_1^{(\mathrm{c})*}, X_2^{(\mathrm{c})*} \in \{-0.3, -0.2, -0.1, 0.0, 0.1, 0.2, 0.3\}$, which essentially moves the void throughout the square matrix. The absolute error of parameter estimation is shown in Fig. S8, with the three panels for $X_1^{(\mathrm{c})}$, $X_2^{(\mathrm{c})}$, and R, respectively. Despite some difference in the value of error, the PINN can accurately estimate the three unknown parameters for all the locations of the void. Consequently, our method is robust about the location of the void.

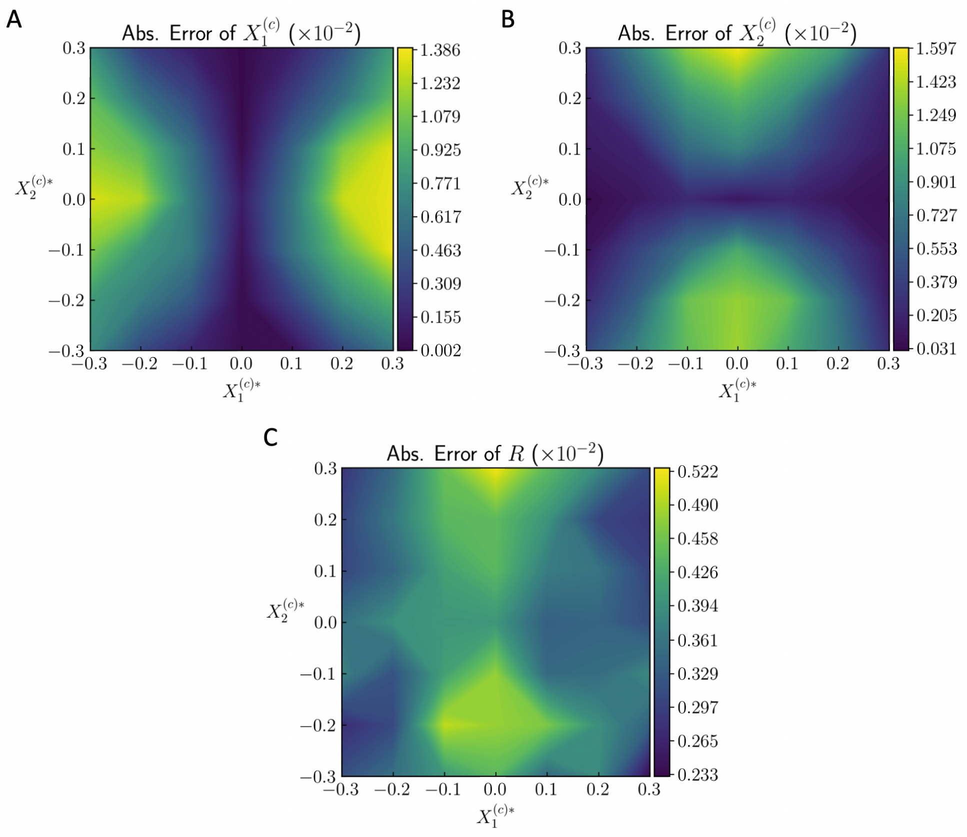

Fig. S8. Parametric study on the location of the void. We show the absolute error of parameter estimation for the three unknown parameters $(X_1^{(\text{c})}, X_2^{(\text{c})}, R)$ for different true locations of the void $(X_1^{(\text{c})*}, X_2^{(\text{c})*})$.

Section S10. Parametric study on Simplified Case 5: Influence of the Moduli Ratio

Here we study the influence of the moduli ratio of the inclusion to the matrix (μ_i/μ_m) on the estimation accuracy. The circular inclusion with known radius $R = 0.2$ is placed at the center of the matrix. The modulus of the matrix $\mu_m = 0.333$ is known to the PINN, and the only unknown parameter is the modulus of the inclusion ($\boldsymbol{\theta}_{\text{unk}} = \mu_i$). We set the moduli ratio μ_i^*/μ_m to range from 0.2 to 5.0, and initialize the modulus of the inclusion to be $\mu_i^0 = 1.5\mu_i^*$ for each case. For each μ_i^*/μ_m, we run the training process until the estimated value converges evidently. Other technical details are the same as case 5 in the main text.

The results of the (signed) relative error for each μ_i^*/μ_m are shown in Fig. S9. Within the range we consider ($0.2 \leq \mu_i^*/\mu_m \leq 5.0$), all cases produce very accurate results, with relative error no larger than 10^{-2}. Therefore, for the prototypical problem, our approach provides accurate estimation results for the moduli ratio ranging from 0.2 to 5.0, which is reasonably large and covers more than one order of magnitude.

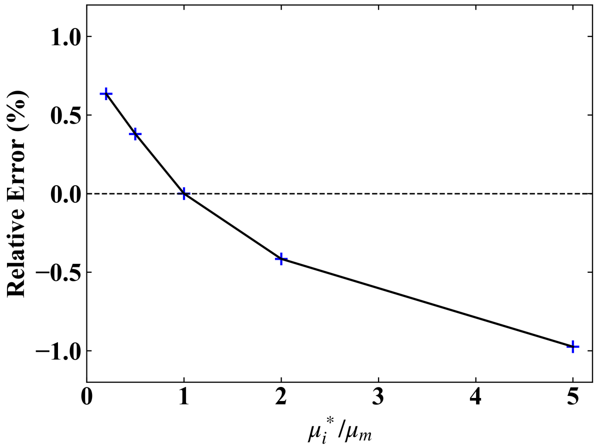

Fig. S9. Parametric study on the moduli ratio of the matrix to the inclusion. We show the (signed) relative error of the estimation of the moduli ratio for different values of μ_i^*/μ_m.

Section S11. L-BFGS Optimizer

Here we do a simple comparison on the model performance in terms of accuracy and efficiency with different strategies on optimizers based on case 4. In the main text, we adopt the Adam optimizer as the only optimizer throughout the entire training process with 1M iterations (called strategy 1A in this section), after which both the parameter estimations and loss function reach a relative plateau. Such a strategy gives high accuracy and helps us study the convergence history as a fundamental characteristic of our method. To achieve a reasonable accuracy practically, one may not need as many as 1M iterations. Here, we consider training the PINN over 200K iterations only (called strategy 1B). To further improve the computational efficiency, we also consider using Adam for the first few iterations (40K iterations in our case), and then switch-

ing to L-BFGS (*48*) (called strategy 2), which is common for training PINNs practically. We compare the results of the three strategies (1A, 1B and 2) in Table S1 in terms of accuracy of parameter estimation and computational efficiency. The computational time is measured by running the code on typical machines using CPU only. The results of strategy 1B indicates that training the PINN with 200K iterations provides reasonably high accuracy. Strategy 2 using L-BFGS performs even better – accuracy similar to strategy 1A is achieved, while the computational cost is significantly reduced.

**Case 4**	$X_1^{(1)}$	$X_2^{(1)}$	$R^{(1)}$	$X_1^{(2)}$	$X_2^{(2)}$	$R^{(2)}$
Reference Value	-0.15	0.10	0.20	0.25	-0.05	0.15
**Strategy 1A: Adam 1M (around 667 minutes)**						
Estimated Value	-0.15089	0.10018	0.20007	0.25045	-0.05008	0.15019
Absolute Error($\times 10^{-2}$)	0.09	0.02	0.01	0.05	0.01	0.02
Relative Error(%)	0.09	0.02	0.04	0.05	0.01	0.13
**Strategy 1B: Adam 200K (around 133 minutes)**						
Estimated Value	-0.15517	0.10191	0.19911	0.24975	-0.05033	0.15298
Absolute Error($\times 10^{-2}$)	0.52	0.19	0.09	0.03	0.03	0.30
Relative Error(%)	0.52	0.19	0.45	0.10	0.03	1.99
**Strategy 2: Adam 40K and L-BFGS (around 35 minutes)**						
Estimated Value	-0.15110	0.10002	0.19998	0.24993	-0.04953	0.15076
Absolute Error($\times 10^{-2}$)	0.11	0.00	0.00	0.01	0.05	0.08
Relative Error(%)	0.11	0.00	0.01	0.01	0.05	0.51

Table S1. Parameter estimation results for case 4 with different strategies on optimizers.

REFERENCES AND NOTES

1. Y. LeCun, Y. Bengio, G. Hinton, Deep learning. *Nature* **521**, 436–444 (2015).

2. A. Krizhevsky, I. Sutskever, G. E. Hinton, ImageNet classification with deep convolutional neural networks. *Adv. Neural Inf. Proces. Syst.* **25**, 1097–1105 (2012).

3. G. Hinton, L. Deng, D. Yu, G. Dahl, A.-r. Mohamed, N. Jaitly, A. Senior, V. Vanhoucke, P. Nguyen, B. Kingsbury, Deep neural networks for acoustic modeling in speech recognition. *IEEE Signal Process. Mag.* **29**, 82–97 (2012).

4. K. T. Butler, D. W. Davies, H. Cartwright, O. Isayev, A. Walsh, Machine learning for molecular and materials science. *Nature* **559**, 547–555 (2018).

5. Z. Shi, E. Tsymbalov, M. Dao, S. Suresh, A. Shapeev, J. Li, Deep elastic strain engineering of bandgap through machine learning. *Proc. Natl. Acad. Sci. U.S.A.* **116**, 4117–4122 (2019).

6. Z. Shi, M. Dao, E. Tsymbalov, A. Shapeev, J. Li, S. Suresh, Metallization of diamond. *Proc. Natl. Acad. Sci. U.S.A.* **117**, 24634–24639 (2020).

7. L. Lu, M. Dao, P. Kumar, U. Ramamurty, G. E. Karniadakis, S. Suresh, Extraction of mechanical properties of materials through deep learning from instrumented indentation. *Proc. Natl. Acad. Sci. U.S.A.* **117**, 7052–7062 (2020).

8. Y.-J. Cha, W. Choi, O. Büyüköztürk, Deep learning-based crack damage detection using convolutional neural networks. *Comput. Aided Civ. Inf. Eng.* **32**, 361–378 (2017).

9. H. Adeli, Neural networks in civil engineering: 1989–2000. *Comput. Aided Civ. Inf. Eng.* **16**, 126–142 (2001).

10. M. Yin, E. Ban, B. V. Rego, E. Zhang, C. Cavinato, J. D. Humphrey, G. E. Karniadakis, Simulating progressive intramural damage leading to aortic dissection using an operator-regression neural network. arXiv:2108.11985 [cs.CE] (25 August 2021).

11. H. Jin, Big-data-driven multi-scale experimental study of nanostructured block copolymer’s dynamic toughness, Ph.D. thesis, Brown University, Providence, RI (2021).

12. M. Raissi, P. Perdikaris, G. E. Karniadakis, Physics-informed neural networks: A deep learning framework for solving forward and inverse problems involving nonlinear partial differential equations. *J. Comput. Phys.* **378**, 686–707 (2019).

13. M. Raissi, A. Yazdani, G. E. Karniadakis, Hidden fluid mechanics: Learning velocity and pressure fields from flow visualizations. *Science* **367**, 1026–1030 (2020).

14. S. Cai, H. Li, F. Zheng, F. Kong, M. Dao, G. E. Karniadakis, S. Suresh, Artificial intelligence velocimetry and microaneurysm-on-a-chip for three-dimensional analysis of blood flow in physiology and disease. *Proc. Natl. Acad. Sci. U.S.A.* **118**, e2100697118 (2021).

15. S. Cai, Z. Mao, Z. Wang, M. Yin, G. E. Karniadakis, Physics-informed neural networks (pinns) for fluid mechanics: A review. arXiv:2105.09506 [physics.flu-dyn] (20 May 2021).

16. E. Samaniego, C. Anitescu, S. Goswami, V. M. Nguyen-Thanh, H. Guo, K. Hamdia, X. Zhuang, T. Rabczuk, An energy approach to the solution of partial differential equations in computational mechanics via machine learning: Concepts, implementation and applications. *Comput. Methods Appl. Mech. Eng.* **362**, 112790 (2020).

17. C. Rao, H. Sun, Y. Liu, Physics-informed deep learning for computational elastodynamics without labeled data. *J. Eng. Mech.* **147**, 04021043 (2021).

18. E. Zhang, M. Yin, G. E. Karniadakis, Physics-informed neural networks for nonhomogeneous material identification in elasticity imaging. arXiv:2009.04525 [cs.LG] (2 September 2020).

19. J. N. Fuhg, N. Bouklas, The mixed deep energy method for resolving concentration features in finite strain hyperelasticity. arXiv:2104.09623 [cs.CE] (15 April 2021).

20. K. Shukla, P. C. Di Leoni, J. Blackshire, D. Sparkman, G. E. Karniadakis, Physics-informed neural network for ultrasound nondestructive quantification of surface breaking cracks. *J. Nondestruct. Eval.* **39**, 61 (2020).

21. M. Yin, X. Zheng, J. D. Humphrey, G. E. Karniadakis, Non-invasive inference of thrombus material properties with physics-informed neural networks. *Comput. Methods Appl. Mech. Eng.* **375**, 113603 (2021).

22. S. Goswami, M. Yin, Y. Yu, G. Karniadakis, A physics-informed variational deeponet for predicting the crack path in brittle materials. arXiv:2108.06905 [cs.LG] (16 August 2021).

23. L. Yang, D. Zhang, G. E. Karniadakis, Physics-informed generative adversarial networks for stochastic differential equations. *SIAM J. Sci. Comput.* **42**, A292–A317 (2020).

24. G. Pang, L. Lu, G. E. Karniadakis, fPINNs: Fractional physics-informed neural networks. *SIAM J. Sci. Comput.* **41**, A2603–A2626 (2019).

25. T. L. Anderson, *Fracture Mechanics: Fundamentals and Applications* (CRC Press, 2017).

26. S. Suresh, *Fatigue of Materials* (Cambridge Univ. Press, 2012).

27. L. B. Freund, S. Suresh, *Thin Film Materials: Stress, Defect Formation and Surface Evolution* (Cambridge Univ. Press, 2010).

28. H. B. Ameur, M. Burger, B. Hackl, Level set methods for geometric inverse problems in linear elasticity. *Inverse Probl.* **20**, 673–696 (2004).

29. H. Sun, H. Waisman, R. Betti, Nondestructive identification of multiple flaws using xfem and a topologically adapting artificial bee colony algorithm. *Int. J. Numer. Methods Eng.* **95**, 871–900 (2013).

30. R. Gallego, G. Rus, Identification of cracks and cavities using the topological sensitivity boundary integral equation. *Comput. Mech.* **33**, 154–163 (2004).

31. J. Jung, E. Taciroglu, Modeling and identification of an arbitrarily shaped scatterer using dynamic xfem with cubic splines. *Comput. Methods Appl. Mech. Eng.* **278**, 101–118 (2014).

32. H. Waisman, E. Chatzi, A. W. Smyth, Detection and quantification of flaws in structures by the extended finite element method and genetic algorithms. *Int. J. Numer. Methods Eng.* **82**, 303–328 (2010).

33. D. Schnur, N. Zabaras, An inverse method for determining elastic material properties and a material interface. *Int. J. Numer. Methods Eng.* **33**, 2039–2057 (1992).

34. Z. Michalewicz, M. Schoenauer, Evolutionary algorithms for constrained parameter optimization problems. *Evol. Comput.* **4**, 1–32 (1996).

35. S. Chaabane, M. Masmoudi, H. Meftahi, Topological and shape gradient strategy for solving geometrical inverse problems. *J. Math. Anal. Appl.* **400**, 724–742 (2013).

36. Y. Mei, R. Fulmer, V. Raja, S. Wang, S. Goenezen, Estimating the non-homogeneous elastic modulus distribution from surface deformations. *Int. J. Solids Struct.* **83**, 73–80 (2016).

37. S. Amstutz, I. Horchani, M. Masmoudi, Crack detection by the topological gradient method. *Control. Cybern.* **34**, 81–101 (2005).

38. T. J. Hughes, *The Finite Element Method: Linear Static and Dynamic Finite Element Analysis* (Courier Corporation, 2012).

39. C. R. Vogel, *Computational Methods for Inverse Problems* (SIAM, 2002).

40. A. Düster, J. Parvizian, Z. Yang, E. Rank, The finite cell method for three-dimensional problems of solid mechanics. *Comput. Methods Appl. Mech. Eng.* **197**, 3768–3782 (2008).

41. A. D. Jagtap, E. Kharazmi, G. E. Karniadakis, Conservative physics-informed neural networks on discrete domains for conservation laws: Applications to forward and inverse problems. *Comput. Methods Appl. Mech. Eng.* **365**, 113028 (2020).

42. Y. Chen, L. Lu, G. E. Karniadakis, L. Dal Negro, Physics-informed neural networks for inverse problems in nano-optics and metamaterials. *Opt. Express* **28**, 11618–11633 (2020).

43. M. Abadi, A. Agarwal, P. Barham, E. Brevdo, Z. Chen, C. Citro, G. S. Corrado, A. Davis, J. Dean, M. Devin, S. Ghemawat, I. Goodfellow, A. Harp, G. Irving, M. Isard, Y. Jia, R. Jozefowicz, L. Kaiser, M. Kudlur, J. Levenberg, D. Mané, R. Monga, S. Moore, D. Murray, C. Olah, M. Schuster, J. Shlens, B. Steiner, I. Sutskever, K. Talwar, P. Tucker, V. Vanhoucke, V. Vasudevan, F. Viégas, O. Vinyals, P. Warden, M. Wattenberg, M. Wicke, Y. Yu, X. Zheng, TensorFlow: Large-scale machine learning on heterogeneous distributed systems (2016).

44. S. Wang, P. Perdikaris, Deep learning of free boundary and Stefan problems. *J. Comput. Phys.* **428**, 109914 (2021).

45. O. Hennigh, S. Narasimhan, M. A. Nabian, A. Subramaniam, K. Tangsali, M. Rietmann, J. d. A. Ferrandis, W. Byeon, Z. Fang, S. Choudhry, NVIDIA SimNet: An AI-accelerated multi-physics simulation framework. arXiv:2012.07938 [physics.flu-dyn] (14 December 2020).

46. Abaqus, *Abaqus 2020 Documentation* (Dassault Systèmes, 2020).

47. D. P. Kingma, J. Ba, Adam: A method for stochastic optimization. arXiv:1412.6980 [cs.LG] (22 December 2014).

48. D. C. Liu, J. Nocedal, On the limited memory BFGS method for large scale optimization. *Math. Program.* **45**, 503–528 (1989).

49. K. Shukla, A. D. Jagtap, G. E. Karniadakis, Parallel physics-informed neural networks via domain decomposition. arXiv:2104.10013 [cs.DC] (20 April 2021).

50. Y. Shin, J. Darbon, G. E. Karniadakis, On the convergence of physics informed neural networks for linear second-order elliptic and parabolic type pdes. arXiv:2004.01806 [math.NA] (2 April 2020).

51. S. Wang, X. Yu, P. Perdikaris, When and why PINNS fail to train: A neural tangent kernel perspective. *J. Comput. Phys.* **449**, 110768 (2020).

52. S. Wang, H. Wang, P. Perdikaris, On the eigenvector bias of Fourier feature networks: From regression to solving multi-scale PDES with physics-informed neural networks. *Comput. Methods Appl. Mech. Eng.* **384**, 113938 (2021).

53. M. M. Doyley, Model-based elastography: A survey of approaches to the inverse elasticity problem. *Phys. Med. Biol.* **57**, R35–R73 (2012).

54. T. D'orazio, M. Leo, A. Distante, C. Guaragnella, V. Pianese, G. Cavaccini, Automatic ultrasonic inspection for internal defect detection in composite materials. *NDT E Int.* **41**, 145–154 (2008).

55. B. Lahiri, S. Bagavathiappan, P. Reshmi, J. Philip, T. Jayakumar, B. Raj, Quantification of defects in composites and rubber materials using active thermography. *Infrared Phys. Technol.* **55**, 191–199 (2012).

56. D.-G. Park, C. S. Angani, B. Rao, G. Vértesy, D.-H. Lee, K.-H. Kim, Detection of the subsurface cracks in a stainless steel plate using pulsed eddy current. *J. Nondestruct. Eval.* **32**, 350–353 (2013).

57. L. Cheng, G. Y. Tian, Surface crack detection for carbon fiber reinforced plastic (cfrp) materials using pulsed eddy current thermography. *IEEE Sensors J.* **11**, 3261–3268 (2011).

58. M. Bashkansky, M. Duncan, M. Kahn, D. Lewis, J. Reintjes, Subsurface defect detection in ceramics by high-speed high-resolution optical coherent tomography. *Opt. Lett.* **22**, 61–63 (1997).

59. M. Klemm, J. Leendertz, D. Gibbins, I. Craddock, A. Preece, R. Benjamin, Microwave radar-based breast cancer detection: Imaging in inhomogeneous breast phantoms. *IEEE Antennas Wirel. Propag. Lett.* **8**, 1349–1352 (2009).

60. L. Lu, R. Pestourie, W. Yao, Z. Wang, F. Verdugo, S. G. Johnson, Physics-informed neural networks with hard constraints for inverse design. arXiv:2102.04626 [physics.comp-ph] (9 February 2021).

61. J. Céa, S. Garreau, P. Guillaume, M. Masmoudi, The shape and topological optimizations connection. *Comput. Methods Appl. Mech. Eng.* **188**, 713–726 (2000).

62. S. Goenezen, P. Barbone, A. A. Oberai, Solution of the nonlinear elasticity imaging inverse problem: The incompressible case. *Comput. Methods Appl. Mech. Eng.* **200**, 1406–1420 (2011).

63. A. D. Jagtap, K. Kawaguchi, G. E. Karniadakis, Adaptive activation functions accelerate convergence in deep and physics-informed neural networks. *J. Comput. Phys.* **404**, 109136 (2020).

64. X. Glorot, Y. Bengio, Understanding the difficulty of training deep feedforward neural networks, in *Proceedings of the Thirteenth International Conference on Artificial Intelligence and Statistics* (PMLR, 2010), pp. 249–256.
